# Regulation of p53 by E3s

**DOI:** 10.3390/cancers13040745

**Published:** 2021-02-11

**Authors:** Mengwu Pan, Christine Blattner

**Affiliations:** Institute of Biological and Chemical Systems—Biological Information Processing, Karlsruhe Institute of Technology, PO-box 3640, 76021 Karlsruhe, Germany; mengwu.pan@kit.edu

**Keywords:** p53, E3s, tumor

## Abstract

**Simple Summary:**

The p53 protein is a transcription factor that initiates cell cycle arrest and apoptosis and by this counteracts tumorigenesis. Because of its anti-proliferative activity, p53 levels are usually low as the protein is rapidly degraded, unless its anti-tumoral activity is required. E3s play an important role in this process. While at earlier times only E3s that target p53 for degradation had been identified, more recent years showed that E3s also control p53 localization and its activity, even independently of its degradation. In addition, more and more E3s that target p53 have been identified in the last years. With this review, we want to provide an overview about the E3s that target p53 and how they control p53 abundance and activity.

**Abstract:**

More than 40 years of research on p53 have given us tremendous knowledge about this protein. Today we know that p53 plays a role in different biological processes such as proliferation, invasion, pluripotency, metabolism, cell cycle control, ROS (reactive oxygen species) production, apoptosis, inflammation and autophagy. In the nucleus, p53 functions as a bona-fide transcription factor which activates and represses transcription of a number of target genes. In the cytoplasm, p53 can interact with proteins of the apoptotic machinery and by this also induces cell death. Despite being so important for the fate of the cell, expression levels of p53 are kept low in unstressed cells and the protein is largely inactive. The reason for the low expression level is that p53 is efficiently degraded by the ubiquitin-proteasome system and the vast inactivity of the tumor suppressor protein under normal growth conditions is due to the absence of activating and the presence of inactivating posttranslational modifications. E3s are important enzymes for these processes as they decorate p53 with ubiquitin and small ubiquitin-like proteins and by this control p53 degradation, stability and its subcellular localization. In this review, we provide an overview about E3s that target p53 and discuss the connection between p53, E3s and tumorigenesis.

## 1. Introduction

**Figure 1:**
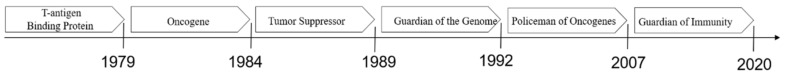
Timeline for the classified roles of p53. In 1979, p53 was identified as a SV40 T-antigen binding protein. Subsequent work suggested p53 to be an oncogene. Ten years after its discovery, it became clear that wild-type p53 functions as a tumor suppressor protein and not as an oncogene. A few years later, p53 was assigned the title “guardian of the genome”. In 2007 the title “policeman of oncogenes” and in 2020 the title “guardian of immunity” were attributed to p53.

p53 was identified 40 years ago by three independent research groups as a protein with a molecular weight of 53 kD that co-precipitated with the SV40 large T antigen in virally-transformed cells [1,2,3]. In the following years, different roles were assigned to p53 (Figure 1). Right after its discovery, p53 was considered to be an oncogene as the protein was only detected in immortalized and transformed mouse fibroblasts and in tumor cells and p53 clones cooperated with oncogenic Ras in cell transformation [3,4,5,6]. It took several years until it was discovered that most of those p53 clones expressed mutated p53 and that wild-type p53 was actually a bona-fide tumor suppressor protein [7]. Further research established that p53 is activated by DNA damage and maintains genomic integrity. Therefore, it was named “guardian of the genome” [8]. About twenty years after its discovery, it was established that p53 also suppresses transformation of cells after activation of oncogenes which brought it the attribute “policeman of oncogenes” [9,10]. Another fifteen years later, it emerged that p53 is also a critical player in normal and cancer immunity [11]. After that, it was also named “guardian of immunity” [12]. During these years, more than one hundred thousand papers where published providing insight into p53 function and regulation.

## 2. The Tumor Suppressor Protein p53

**Figure 2:**
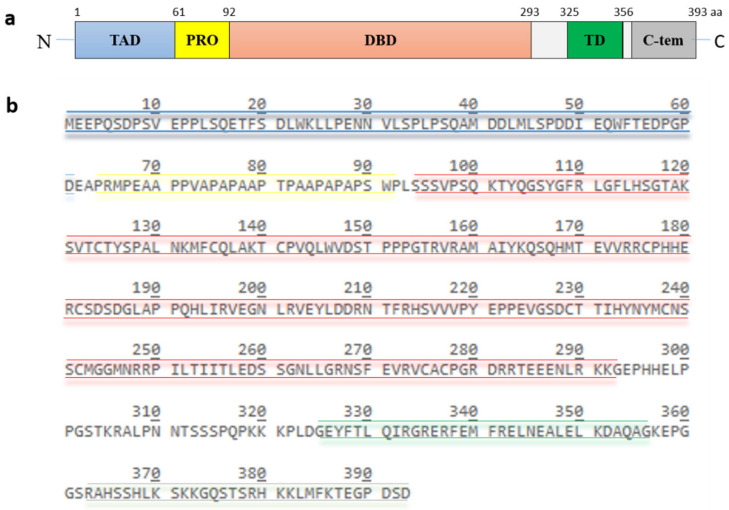
Structure and amino acid sequence of p53. (**a**) p53 possesses five highly conserved domains: a N-terminal transactivation domain (TAD), a proline-rich domain (PRO), a central DNA-binding domain (DBD), a tetramerization domain (TD) and a C-terminal basic domain. (**b**) the amino acid sequence of the different domains is highlighted in color. The TAD, consisting of residues 1 to 61, is colored in blue, the PRO, consisting of residues 64 to 92, is colored in yellow, the DBD, consisting of residues 94 to 293, is colored in red, the TD, consisting of residue 325 to 356, is colored in green and the C-terminus, consisting of residues 363 to 393, is colored in grey.

p53, encoded by the TP53 gene, is a protein of 393 amino acids (390 amino acids in the case of murine p53) and contains five highly conserved domains (Figure 2): a N-terminal transactivation domain, a proline-rich region, a central DNA binding domain, a tetramerization domain and a C-terminal basic domain [13]. The transactivation domain is required for transcriptional activation of p53 target genes. It is normally unfolded and a site of intensive protein-protein interactions. Components of the transcriptional machinery such as TFIID (transcription factor IID) and TFIIH (transcription factor IIH), histone acetyltransferases like p300 and CBP (CREB-binding protein), and also the E3 MDM2 (mouse double minute 2) bind to this site [14,15,16,17,18,19,20]. The proline-rich region was initially regarded as a spacer between the transactivation domain and the DNA-binding domain. It contains five PXXP (proline-XX-proline, where X is any amino acid) motifs which are required for multiple protein-protein interaction and for transcriptional activation and repression [21]. The DNA binding domain is a core functional domain of p53. It binds to p53 consensus DNA-binding sites on target genes [22]. The tetramerization domain allows the formation of a dimer. Two dimers of p53 form a homotetramer, which is the transcriptionally active form of p53 [23]. The C-terminal basic domain is also intrinsically disordered and a site of various post-translational modifications including ubiquitination, phosphorylation, acetylation, methylation, sumoylation and neddylation [24,25,26,27,28].

p53 is best known for the induction of cell cycle arrest and apoptosis upon its activation. Studies from the early 1990s show that p53 is a key element for the reversible DNA damage-induced G1/S checkpoint [29]. This checkpoint is controlled by the cyclin-dependent kinase inhibitor p21, one of the first identified target genes of p53 [30,31]. The apoptosis-inducing function of p53 relies on the induction of genes like Bax, Puma or Noxa and the repression of BCL-2 [32,33,34]. Under normal growth conditions, p53 is largely inactive. However, when cells experience an increased risk for transformation, p53 is protected from degradation. How p53 is protected from degradation depends on the applied stress signal. Ionizing irradiation leads to the activation of the ataxia telangiectasia mutated (ATM) kinase and the DNA damage-dependent protein kinase (DNA-PK). Both kinases are at the beginning of signal transduction cascades leading to phosphorylation of the N-terminus of p53 and the C-terminus of MDM2, and dephosphorylation of the central domain of MDM2, resulting in a weakening of the interaction of p53 and MDM2 and inhibition of MDM2 activity [35,36,37]. Irradiation with UV-light also leads to phosphorylation of p53 and MDM2 and to downregulation of MDM2 levels. Seven serine residues (S6, S9, S15, S20, S33, S37 and S46) and two threonine residues (T18, T81) of p53 and six serine residues (S386, S395, S407, S419, S425 and S42) and three tyrosine residues (Y276, Y394 and Y405) of MDM2 can be phosphorylated by ATM, ATR (ATM-related) DNA-PK, Chk1 (checkpoint kinase 1), Chk2, casein kinase 1 and c-abl in response to DNA damage [35,36]. Activation of ATM after ionizing irradiation also leads to the inactivation of other E3s for p53. Phosphorylation of COP1 on serine S387 and of TRIM24 on serine 768 leads to the degradation of the E3s, disruption of the p53/E3 complex, inhibition of p53 ubiquitination and stabilization of p53 [37,38]. Oncogenes such as Myc or Ras also lead to p53 stabilization but they use a different route. They induce expression of p14/16^ARF^ which binds to MDM2, inhibits its ubiquitin ligase activity, sequesters MDM2 in the nucleolus and promotes MDM2 degradation [10,39,40,41,42].

A growing body of work implicates that p53 is also involved in non-canonical programs which affect cellular processes such as protein translation, ROS production, autophagy, epithelial-mesenchymal-transition (EMT), cellular metabolism and control of pluripotency among others [43,44,45]. Although p53 controls a broad network of activities, expression levels of p53 in healthy unstressed cells are usually low and the residual p53 protein is largely inactive. The major reason for the low level of p53 is that the ubiquitin-proteasome system efficiently degrades p53 and p53 lacking activating post-translational modifications is largely inactive.

## 3. E3s and The Ubiquitin Proteasome System

**Figure 3:**
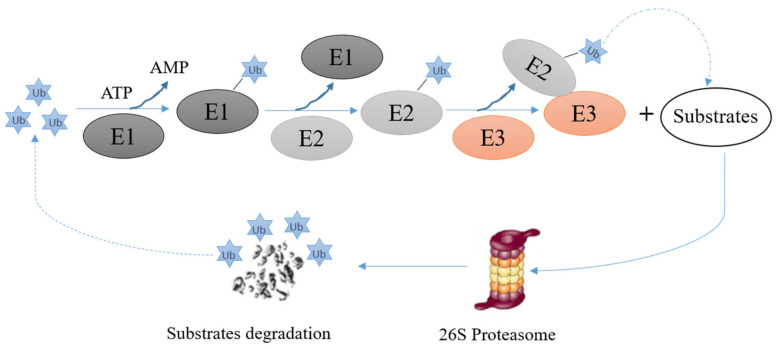
Schematic drawing of protein ubiquitination and degradation. Firstly, ubiquitin molecules are activated by an E1 in an ATP-dependent manner; then the activated ubiquitin molecule is transferred to an E2. In a next step, the E2/Ubiquitin complex binds to an E3. The E3 mediates p53-ubiquitination and promotes degradation of p53 in 26S proteasomes.

E3s are critical elements of the ubiquitin proteasome system. They mediate the last step of the ubiquitination reaction and confer specificity and efficiency to the ubiquitination reaction. The ubiquitination reaction consists of the following steps: (i) the ubiquitously expressed protein ubiquitin binds to a ubiquitin activating enzyme, also called E1, and becomes activated in an ATP-dependent manner, (ii) the activated ubiquitin is transferred from the E1 to an ubiquitin conjugating enzyme, also called E2, (iii) an ubiquitin ligase, also called E3 mediates the transfer of activated ubiquitin to a specific target protein. Depending on the properties of this E3, the E3 may directly transfer the ubiquitin protein to the target protein or may provide a scaffold to bring the E2 and the substrate into an orientation that allows the transfer of ubiquitin from the E2 to the substrate. During repeated rounds of ubiquitination, ubiquitin moieties are attached to the ubiquitin protein(s) that are already attached to the target protein, resulting in a ubiquitin chain appended to the target protein (Figure 3). After a minimum of four rounds of the ubiquitination cycle, proteins can enter 26S proteasomes where they are degraded [46] (Figure 3). For some proteins, including p53, an additional enzyme, a so called E4, is occasionally required, probably to assist for a higher order organization of the polyubiquitin chain [47].

As ubiquitin itself possesses seven lysine residues (K6, K11, K27, K29, K33, K48 and K68) and as each of these lysine residues, together with the first methionine (Met1), can be used for chain formation, a multitude of different chains are possible, particularly as not only homotypic ubiquitin chains, where only one lysine of different ubiquitin molecules is used for chain formation are possible, but also heterotypic chains, where different lysine residues of different ubiquitin molecules are used. Polyubiquitin chains can, furthermore, be linear or branched. Apart from being modified with ubiquitin chains, proteins can also be modified with individual ubiquitin proteins at several sites, resulting in multi-monoubiquitination, or with only one ubiquitin, resulting in monoubiquitination. In addition, chains can be formed with different proteins of the ubiquitin family (ubiquitin, SUMO, NEDD8 etc.), resulting in mixed chains [48,49]. According to the structure and composition of the chain, different functions have been found associated with the modification. Monoubiquitination appears to predominantly modulate activity, trafficking, and interactions of substrates and is less frequently involved in protein degradation, although it seems to be able to promote degradation of some short-lived and eventually of small and loosely-folded proteins [49]. Met1 linked chains are best understood in the context of NF_k_B signaling, inflammation and immunity. The role of lysine K6-linked chains is less clear, but as such chains do not accumulate after proteasome inhibition, they are most likely not involved in protein degradation. They have, however, been shown to accumulate after UV-irradiation, suggesting that they might be involved in the DNA damage response. In addition, they have been linked to mitochondrial homeostasis and mitophagy [48]. Lysine K11-linked chains are relatively abundant and typically found in mixed or branched chains together with lysine K48- and K63-linked chains and can also promote protein degradation, particularly in the context of cell cycle control. Whether homotypic lysine K11 chains are involved in protein degradation is still debated [48,49]. The role of lysine K27-linked chains is not well understood, but evidence suggests that they might be involved in the recruitment of binding partners [48]. Lysine K29-linked chains accumulated after proteasome inhibition, suggesting that they might be involved in protein degradation. Other evidences suggest that they might be involved in the regulation of the proteasome [48]. Lysine K33-linked chain have been found to be involved in post-Golgi membrane protein trafficking, while Lysine K48-linked chains are the most common chains and the prototype for proteasomal degradation [48,49]. Lysine K63-linked chains seem to be involved in the DNA damage response, but they may also collaborate with K6-, K11- and K48-linkages and mediate degradation of damaged mitochondria through mitophagy [49]. Lysine K63-linked chains have also been found in combination with Met1 linked-chains in inflammation and NF_k_B signaling [48]. Branched chains have been found to initiate endocytosis or lead to non-degradable ubiquitin structures and proteasome stalling [48].

Depending on their structural elements, E3s are subdivided into ten classes: (i) RING (really interesting new gene)-domain E3, (ii) HECT (homologous to E6AP carboxyl terminus)-domain ligases, (iii) U-box type E3s (iv) F-box-type E3s, (v) SOCS-box-type E3s, (vi) BTB-type E3s, (vii) DDB1-like E3s, (viii) ZnF A20-like E3s, (ix) the MALT1/paracaspase E3 and (x) RBR (RING-InBetween-RING)-type E3s [50,51,52].

Most E3s lack catalytic activity and are unable to catalyze the formation of peptide bonds. These E3s merely serve as scaffolds for protein-protein interactions. RING-type E3s, for instance, induce, upon interaction with a suitable E2, an allosteric change in the protein conformation of the E2 resulting in the discharge of the ubiquitin protein from the E2 [50]. HECT-type E3s, in contrast, possess catalytic activity. They first form a thioester-linked intermediate with ubiquitin and then transfer the ubiquitin to the substrate [53]. U-box type E3s mediate poly-ubiquitination of their substrates in the absence of a RING or HECT domain. Similar to RING-type E3s, U-box E3s merely serve as scaffolds to allow the transfer of ubiquitin from an E2 to the substrates without forming a U-box/ubiquitin intermediate [54]. The RBR-type E3s are a newly discovered class of E3s with two predicted RING domains, RING1 and RING2 and a central zinc-binding domain between the RINGs. They are regarded as a hybrid of RING and HECT-like domain E3s and can directly catalyze the transfer of ubiquitin to the target protein via their HECT-like domain while they recruit an E2 with one of the RING domains. [51].

With the availability of the sequences of the human genome, more than six hundred putative E3s were annotated [52]. The largest group of these E3s are RING-domain E3s with about three hundred members. Much less frequent are BTB-type E3s with one hundred sixty-nine members and F-box-type E3s with sixty-one member [52]. Relevant for p53 are only five of the ten classes of E3s, namely RING-domain E3s, HECT-domain E3s, U-box-type E3s, an F-Box E3 and an RBR-type E3.

## 4. E3s That Target Wild-Type p53

### 4.1. E3s That Ubiquitinate p53 and Target. It for Degradation

**Figure 4:**
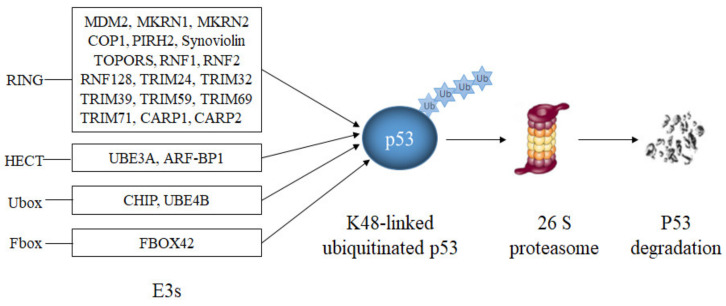
E3s that target p53 for degradation. RING-domain E3s (MDM2, Synoviolin, MKRN1, MKRN2, COP1, PIRH2, TOPORS, CARP1, CARP2, RNF1, RNF2, RNF128, TRIM24, TRIM32, TRIM39, TRIM59 and TRIM71), a HECT-domain E3 (ARF-BP1), two U-Box-domain E3s (CHIP, UBE4B) and a SCF-complex with the F-Box-domain protein FBXO42 modify p53 with lysine K48-linked polyubiquitin chains resulting in p53 degradation.

Most E3s that have been reported to modify p53 target p53 for proteasomal degradation (Table 1, Figure 4). The largest group of them are E3s with a RING domain.

#### 4.1.1. RING-Domain E3s

RING-domain E3s are characterized by the presence of a RING (Really Interesting New Gene) domain. The amino acid sequence of the RING normally follows the pattern C-X_2_-C-X_(9-39)_-C-X_(1-3)_-H-X_(2-3)-_C-X_2_-C-X_(4-48)-_C-X_2_-C, also abbreviated as C3HC4 or RING-HC. Two zinc atoms coordinate the conserved seven cysteine residues and the histidine residue and stabilize the structure. Variations of the RING pattern, where a cysteine residue is replaced by a histidine residue as in the RING-H2 variant or where cysteine residues and histidine residues are swapped or replaced by another amino acid that can coordinate zinc, have also been observed [94]. RING-domains are sites of protein-protein interactions, in particular with E2s. Although some E3s act as single proteins, they have a preference to form homo and heterodimers [94].

The most studied RING-type E3 is MDM2. MDM2 binds to the transactivation domain of p53, inhibits its transcriptional activity and mediates p53 ubiquitination and degradation via 26S proteasomes [55,56,57]. Since MDM2 is also a transcriptional target of p53, the two proteins form a negative feedback loop. This feedback loop restrains p53 abundance since as soon as p53 levels rise, MDM2 levels rise as well and foster p53 degradation [58]. MDM2 mediates ubiquitination of p53 with K48-linked polyubiquitin chains at six key lysine residues in the C-terminus (K370, K372, K373, K381, K382 and K386) resulting in p53 degradation [59]. Genetic deletion of MDM2 leads to embryonic death that is rescued by simultaneous deletion of p53, demonstrating that MDM2 is a key regulator of p53 [95]. After DNA damage, p53 is released from MDM2, allowing its accumulation to high levels and transcriptional activation of its target genes [35]. Thirty-seven different isoforms can be generated from the MDM2 gene, although it is unclear whether they are all translated. Most of them lack a p53-binding site. MDM2 isoforms are more frequently expressed in poorly differentiated and late-stage tumors [96,97]. Apart from full-length MDM2, only the smallest isoform (MDM2-E) co-precipitates with p53 [96]. Although the isoform MDM2-A does not bind to p53, it causes p53-dependent perinatal lethality and senescence [97]. As MDM2 forms dimers, the full-length protein can heterodimerize with MDM2 isoforms, allowing the isoforms to control p53 abundance and activity in trans. Some heterodimers consisting of full-length MDM2 and one of its isoforms are even more potent in ubiquitinating p53 than homodimers of the full-length protein [98]. Of note, despite the increase in p53 ubiquitination, heterodimers of MDM2-B and full-length MDM2 even increased p53 activity [99,100]. More recently, it was found that MDM2-C also has E3 activity and ubiquitinates wild-type and mutated (R273H) p53 [101]. However, it was not determined whether this ubiquitination of p53 by MDM-C leads to p53 degradation.

Other RING-domain E3s that mediate p53 degradation are MKRN1 and MKRN2. MKRN1 and MKRN2 are members of the MKRN (Makorin Ring) protein family, a highly conserved but not very well-investigated family of RING-domain containing proteins. MKRN1 mediated polyubiquitination of p53 at lysine residues K291 and K292 resulted in p53 degradation and downregulation of MKRN1 induced p53-dependent cell cycle arrest [60]. Interestingly, MKRN1 is also an E3 for p21 and promotes p21 poly-ubiquitination and degradation. Upon DNA damage, the interaction of p53 and MKRN1 is strongly reduced resulting in elevated p53 levels while degradation of p21 is still ongoing. Therefore, under normal growth conditions, MKRN1 keeps cells alive by suppressing p53 and p21. However, when cells experience cellular stress, MKRN1 primarily induces the degradation of p21 leading to increased cell death [60]. MKRN2 also binds and ubiquitinates p53 resulting in downregulation of the tumor suppressor protein. MKRN2 is highly expressed in human malignant melanoma cell lines and downregulation of MKRN2 inhibited melanoma growth in a p53-dependent manner [61].

COP1 (constitutive photomorphogenic protein 1) is another RING-domain E3 that mediates p53 degradation. COP1 was identified by mass spectrometric analysis as a binding partner of p53. COP1 increased p53 turnover by targeting it for ubiquitin-mediated degradation and deletion of COP1 stabilized p53, inhibited p53-dependent transcription and arrested cells in the G1 phase of the cell cycle. Although COP1 was able to target p53 for degradation by itself, depletion of both, COP1 and MDM2, by siRNA showed that these two E3s can cooperate and sensitize U2OS cells to ionizing-radiation-induced cell death. COP1 is, furthermore, a target gene of p53, which connects the two proteins by a negative feed-back loop [62]. Like Mdm2, COP1 also possesses splice variants. A total of eleven transcripts have been generated from the human COP1 pre-mRNA and shown to exist in human cell lines. Together with the full-length protein, the COP1D variant is the most abundantly expressed isoform and the only one that has been investigated in human cells and tissues. COP1 and COP1D are nuclear proteins. Their expression varies between different cell lines and tissues. Both isoforms are induced by UV-light albeit with different ratios. Full length COP1 and COP1D form homo- and heterodimers. While COP1D reduced the ability of the full-length protein to target c-Jun for degradation, both isoforms decreased p53 levels under steady-state conditions and after UV-exposure [102].

A further RING-E3 for p53 is PIRH2 (p53-induced protein with a RING-H2 domain). PIRH2 was identified as a p53 target with a RING-domain by differential display [63]. Further analysis showed that PIRH2 binds to p53 and that overexpression of PIRH2 reduces p53 levels because of PIRH2-mediated polyubiquitination and degradation. Accordingly, transcriptional and growth inhibitory activities of p53 were also reduced after overexpression of PIRH2 [63]. The overall ligase activity of PIRH2 towards p53 was lower than that of MDM2 and PIRH2 modified a different set of lysine residues [64]. Like MDM2, PIRH2 is a transcriptional target of p53 and was induced after ionizing irradiation in murine embryonic fibroblasts, yet not to the same extent as MDM2. Of note, activation of p53 in MCF-7 and A549 cells by DNA damage did not lead to the accumulation of PIRH2, pointing to a cell type-dependent regulation of PIRH2 [63,65]. PIRH2 is highly expressed in several cancer cell lines regardless of the p53 status, suggesting that it also has p53-independent oncogenic activities [65]. PIRH2 also comes in several isoforms. A total of six isoforms have been described so far (PIRH2A to PIRH2F, where PIRH2A is the full-length protein) [103,104,105]. PIRH2B and PIRH2C have been detected in different cell lines and PIRH2E and PIRH2F in HepG2 cells [103,105]. PIRH2B and PIRH2C show the same diffuse staining in the nucleus and cytoplasm as the full-length protein. While PIRH2B is able to dimerize with PIRH2A, this is not the case for PIRH2C, yet all three proteins co-precipitated with MDM2 [105]. PIRH2B associates with p53 to a similar extent as the full-length protein while PIRH2C shows only weak interactions with p53. Nevertheless, both isoforms were able to ubiquitinate p53, to target it for degradation and to reduce its transcriptional activity. However, because their RING domains are disrupted, neither PIRH2B nor PIRH2C were able to ubiquitinate p53 directly and required other proteins, eventually PIRH2A or MDM2 [105]. PIRH2D lacks the C-terminal RING domain and thus most likely also E3 activity [104].

Synoviolin is a RING-type E3 that is located in the endoplasmatic reticulum (ER). Synoviolin was identified as an E3 for p53 in cells where synoviolin had been genetically deleted and where p53 accumulated. Synoviolin binds directly to p53, sequesters p53 in the ER and mediates ER-associated polyubiquitination and degradation of p53. Knocking-down synoviolin resulted in p53 accumulation and induction of the GADD45 (growth arrest and DNA damage-inducible protein), MDM2 and p21 genes [66]. The activity of synoviolin is regulated by ER stress. In unstressed cells, synoviolin keeps the steady state levels of p53 low by ubiquitinating it for 26S proteasome-dependent degradation. After mild ER-stress, synoviolin is transcriptionally upregulated and the increased amount of synoviolin is still able to target p53 for degradation. However, under severe or prolonged ER-stress, the overwhelming amount of unfolded proteins may squelch and release p53 from synoviolin and allow its accumulation and activation [67].

Another RING-type E3 that mediates p53 degradation is TOPORS (TOP1 Binding Arginine/Serine Rich Protein, E3). TOPORS was initially found as a p53-binding protein by a yeast two hybrid system with p53 as a bait [68]. Overexpression of TOPORS resulted in a proteasome-dependent decrease in p53 protein expression in human osteosarcoma cells. TOPORS can cooperate with the E2 conjugating enzyme, UbcH5a, Ubch5c and UbcH6 for mediating p53 ubiquitination [69]. Ubiquitination and degradation of p53 by TOPORS is regulated by phosphorylation of TOPORS at serine S718 by the Polo-like kinase 1 (PLK1). Plk1-mediated phosphorylation of TOPORS enhances p53 ubiquitination and degradation. As PLK1 expression is highest in late G2 and S-phase of the cell cycle, this is probably also the time when TOPORS-mediated regulation of p53 occurs [70]. TOPORS is induced after DNA damage, however, while several E3s are target genes of p53, the DNA damage-induced induction of TOPORS is independent of p53 [106].

Two more recently discovered E3s for p53 are RNF1 and RNF2, also known as RING1 and RING1B [71,72]. RNF1 is a crucial component of the transcriptional repression complex PRC1 and RNF2, is part of the transcriptional repression complex PRC2 of the polycomb group. RNF1 directly interacts with p53 and mediates its ubiquitination and degradation by 26S proteasomes. Depletion of RNF1 resulted in p53 stabilization and p53-dependent cell cycle arrest, apoptosis and senescence of HepG2 and HCT116 cells. This effect was compromised in p53-deficient cells showing that the induction of apoptosis and senescence occured via p53 and not through other activities of RNF1 [71]. RNF1 is even capable of downregulating p53 during the DNA damage response [71]. RNF2 binds to p53 and mediates p53 ubiquitination and degradation, however, only in selected cells and cell lines including cells from germ cell tumors or ovarian cancers [72,73]. Also, RNF2 requires the Bmi1 protein of the PRC2 complex for full activity [73]. Furthermore, although RNF2 can bind directly to p53, it also binds to MDM2 and forms a ternary complex with the two proteins. Overexpression of RNF2 was not sufficient to mediate p53 ubiquitination in vitro or in HEK293T cells, suggesting that RNF2 may require MDM2, Bmi1 or a further protein to promote p53 ubiquitination [72]. Downregulation of RNF2 primarily leads to the induction of cell cycle arrest-linked target genes of p53 and of genes that contribute to cellular metabolism but not to the induction of the pro-apoptotic target genes Bax, Noxa and Puma [107]. Starvation of cells, which downregulates RNF2 expression, leads to enhanced p53-dependent apoptosis and to liver atrophy in mice [73]. Although RNF1 can form complexes with RNF2, the association of RNF1 with p53 and its ubiquitination and degradation are independent of an interaction with RNF2 [73].

Other RNF proteins that target p53 are RNF38 and RNF128. RNF38 is widely expressed in a variety of human tissues and evolutionary conserved [108]. Its interaction with p53 was first identified in a genome-wide in vitro expression pull-down screen using Drosophila p53 as a bait [109]. RNF38 is a nuclear protein and predicted to have three isoforms. At least the full-length protein interacts with the E2 enzyme UbcH5b and shows E3 activity. The interaction of RNF38 with p53 was confirmed using bacterially expressed proteins, showing that the interaction is direct. RNF38-mediated ubiquitination of p53 was observed in vitro and after overexpression in cells. Overexpression of RNF38 forced p53 into punctate structures in the nucleus, some of them colocalized with PML bodies [110]. Unfortunately, no data are available on p53 stability upon overexpression of RNF38. Thus, it is also possible that RNF38-mediated ubiquitination of p53 leads to its activation which would be consistent with the deletion of the chromosomal area, where the RNF38 gene is located, in several malignancies [110]. RNF128, also known as Grail, is a transmembrane protein that is localized in endosomes and best-known as a regulator of energy and cytokine production [111]. RNF128 usually exists as a tri-molecular complex comprising Grail, Otub1, and USP8 and possesses E3 activity [74]. The interaction of RNF128 with p53 was identified through a yeast two-hybrid SOS recruitment system for identifying p53-interacting proteins. RNF128 interacts with the N-terminal domain of p53 under normal growth conditions as well as after DNA damage. [74]. Overexpression of RNF128 reduced p53 levels and stability, and expression of its downstream target genes p21 and Bax, and p53-dependent apoptosis. RNF128 is a p53 target gene and as it is able to target p53 for degradation after treatment with actinomycin D or exposure to UV-light, it may contribute to the downregulation of p53 after DNA damage, particularly as RNF128 is induced by p53 under these conditions [74]. As RNF128 is highly abundant in heart and liver, it may regulate p53 expression especially in these tissues. Although RNF128 downregulates p53, its expression was significantly reduced in advanced urinary cancer and its low expression was correlated with high tumor stage, high histological grade, high proliferation and vascular and perineural invasion [112].

Some other RING-type E3s that mediate p53 ubiquitination and degradation belong to the TRIM-family of proteins, a protein family with more than 80 members that are characterized by an N-terminal RING-domain, one or two B-Boxes and a coiled-coiled region. Due to the presence of a RING-domain, most TRIM proteins possess E3 activity. TRIM24, TRIM32, TRIM39, TRIM59, TRIM69 and TRIM71 all mediate ubiquitination and degradation of p53 [75,76,77,80,81]. TRIM24 was first identified as a co-repressor of the retinoic acid receptor alpha [113]. Later, by a tandem-affinity-purification approach with subsequent mass spectrometry analysis, it was found to associate with p53 [75]. TRIM24 ubiquitinates p53 and promotes its degradation in 26S proteasomes. Downregulation of TRIM24 in human tumor-derived cancer cells resulted in p53-dependent apoptosis [75]. In contrast to MDM2^−/−^ embryos, which die a few days after fertilization, TRIM24^−/−^ mice are viable [114,115]. However, mutation of the TRIM24 homolog Bonus led in Drosophila to apoptosis that was rescued by p53 deletion [75]. Like many E3s that target p53, TRIM24 is also a target gene of p53, connecting the two proteins by a negative feedback-loop [38]. DNA damage leads to phosphorylation of TRIM24 at serine S768 by the ATM kinase, resulting in the release of p53 from TRIM24 and degradation of TRIM24 [38]. Due to the high levels of p53 after DNA damage, TRIM24 is continuously synthesized and as it can even bind to phosphorylated p53 and target it for degradation, TRIM24 contributes to the return to normal p53 levels once DNA lesions have been repaired [38]. TRIM32 is best known for its activity during neuronal and skeletal muscle cell differentiation [116]. However, the ligase is also frequently overexpressed in head and neck cancers and in skin carcinoma [117]. In line with promoting p53 degradation, TRIM32 has been shown to reduce the transcriptional activation of p53 target genes, both under non-stressed conditions and after DNA damage, and to reduce p53-mediated cell cycle arrest and apoptosis while downregulation of TRIM32 increased expression of p53-target genes in a p53-dependent manner [118]. As TRIM32 is, like MDM2 or TRIM24, a target gene of p53, both proteins are connected by a negative feedback loop [76]. Also, TRIM39, better known as a regulator of the anaphase promoting complex [119], can directly bind to p53, ubiquitinate the tumor suppressor protein and target it for degradation. Most interestingly, cells harboring wild-type p53 cannot traverse the G1/S checkpoint when TRIM39 is absent from the cell and this cell cycle arrest depends strongly on p53 [77]. Although TRIM39-mediated ubiquitination of p53 is independent of MDM2, TRIM39 synergizes with MDM2 to promote cell cycle arrest and apoptosis via promoting p53 degradation. This is possible as both proteins bind to different domains of p53 [77]. TRIM59, attracted attention when it was found to be highly overexpressed in gastric cancer. Its increased levels were, furthermore, associated with more advanced tumor stages and shorter survival times [78]. Knocking-down TRIM59 reduced proliferation and clonogenicity of gastric cancer cells and the growth of xenografts in nude mice while increased levels of TRIM59 are correlated with decreased expression of p53 target genes, probably resulting from the interaction of TRIM59 with p53 and its ubiquitination and degradation [78]. TRIM69 is a protein that is present in the cytoplasm and nucleus where it forms speckled aggregates in a RING-dependent manner [120]. It is highly expressed throughout zebrafish embryogenesis and in most adult tissues with highest expression in testis, brain, heart and ovary [79]. Knocking-down TRIM69 by morpholino-antisense-oligonucleotides induced massive cell death that was rescued by knocking-down p53 [79]. Further analysis showed that overexpression of TRIM69 reduced UVB-induced apoptosis while downregulation of TRIM69 induced it [80]. TRIM69 interacted with p53 and induced its ubiquitination [80]. TRIM69 is downregulated in cataract lenses whereas p53 levels are higher under these conditions, suggesting that the regulation of p53 by TRIM69 might play a role in this process. [80]. TRIM71 is an E3 that is especially linked to stem cell pluripotency, reprogramming and neurogenesis. It is indispensable for neural tube closure and embryonic development. TRIM71 interacts directly with p53, controls its abundance by ubiquitinating lysine residues K313-K315 within the nuclear localization signal and antagonizes p53-dependent pro-apoptotic and pro-differentiation responses [81]. TRIM71^−/−^ cells showed upregulation of Grhl3 and Caspase-3 activation, two downstream effectors of p53 [81]. TRIM71 is particularly active after the onset of differentiation of embryonic stem cells. Overexpression of TRIM71 decreased p53 protein levels in this setting and increased cell proliferation. Most importantly, induction of neural differentiation is associated with p53-dependent accumulation of cleaved Caspase-3 and this is strongly reduced upon overexpression of TRIM71. Loss of TRIM71 resulted in aberrant p53 activation during neural tube closure leading to massive cell death and an unclosed neural tube [81].

Another protein family where members show E3 activity towards p53 are the CARPs (Caspase8/10-associated RING proteins) [82]. CARPs (CARP1 and 2) usually target Caspase 8 and 10 for degradation and are cleaved by the activated Caspases 8 and 10 once the extrinsic cell death pathway is initiated [82]. CARPs are overexpressed in tumors and their downregulation reduced cell viability. CARP1 and 2 physically interacted with p53, ubiquitinated it, promoted its degradation and reduced the expression of p53 target genes and the induction of cell cycle arrest. CARPs belong to the few E3s that also target phosphorylated p53 for degradation and thus contribute to the return to basal p53 levels once DNA lesions are repaired [121]. In addition to the direct role for p53 degradation, CARPs bind to MDM2 and stabilize MDM2 by inhibiting its self-ubiquitination. The resulting elevated levels of MDM2 also contribute to the strong decrease in p53 levels upon overexpression of CARPS [122].

#### 4.1.2. HECT-Domain and U-Box-Domain E3s

Apart from RING-domain containing E3s, some U-Box and HECT-domain containing E3s target p53 for degradation. Among these E3s are the HECT-domain containing proteins UBE3A and ARF-BP1 and the U-box E3s CHIP and UBE4B.

The HECT domain of E3s roughly consists of 350 amino acids that form a larger N-terminal and a smaller C-terminal lobe that are connected by a short linker [123]. The N-terminal lobe is the landing site for the E2 while the C-terminal lobe contains the active-site cysteine that forms a thioester bond with ubiquitin resulting in an E3-ubiquitin intermediate before the ubiquitin is transferred to its substrate [124].

UBE3A was the first HECT-type E3 that was found to be involved in p53 regulation [83,84]. Over-expression of UBE3A in neuro 2a cells increased ubiquitination and degradation of p53 that could be prevented by deletion of the HECT-domain of the ligase. Partial knockdown of UBE3A increased p53 levels and p53-dependent transcription, and also promoted neuronal cell death [85]. In vitro ubiquitination assays showed that UBE3A directly interacts with and ubiquitinates p53 [86]. UBE3A comes in several isoforms. Five mRNA subtypes have been isolated that are all expressed in cells, yet to varying degree, giving rise to three isoforms. Isoform I corresponds to the previously reported open reading frame for E6-AP while isoforms II and III have additional 20 and 23 amino acids, respectively, at their N-termini and utilize different initiation codons [125]. No function has as yet been assigned to the different isoforms.

ARF-BP1 is with approximately 500 kD one of the largest proteins in the cell. It was identified as a protein that co-purified with overexpressed p14/p19^ARF^ protein, the alternative transcript of the Ink4a/ARF tumor suppressor locus [87]. p14/p19^ARF^ exhibits tumor suppressive activities by stabilizing p53 in response to oncogenic stimuli. Oncogenes such as Myc or Ras induce the p14/p19^ARF^ protein resulting in nucleolar sequestration and degradation of MDM2 with the consequence of p53 stabilization and activation [126]. In wild-type cells, ARF-BP1 binds directly to p53 and ubiquitinates p53, an activity that is inhibited by the p14/p19^ARF^ protein [87]. Inactivation of ARF-BP1 is essential for p14/p19^ARF^—mediated stabilization of p53 e.g., in response to oncogenic stimuli [87]. Knock-down of ARF-BP1 also elevates the levels of endogenous p53, increases expression of its target genes and induces cell death. ARF-BP1-mediated cell death is p53-dependent and not seen in p53-negative HCT116 cells. Inactivation of ARF-BP1 during embryonic development resulted in p53-activation and embryonic lethality, however, mice where ARF-BP1 was only deleted in pancreatic β-cells were viable and displayed no obvious abnormality after birth, indicating that the regulation of p53 by ARF-BP1 is particularly powerful in a subset of cells and tissues [88].

The U-box is a domain of about 70 amino acids that is present in proteins from yeast to man. The predicted structure of the U-box is similar to that of the RING domain, yet U-boxes lack the metal coordinating histidine and cysteine residues that are a hallmark of the RING domain [127]. Like RING-domain E3s, U-box E3s lack a catalytic cysteine and rather act as a scaffold to orient the E2 and the target to allow efficient ubiquitination of the target protein. In mammals, there are six U-box proteins that mediate ubiquitination of target genes in conjunction with E1s and E2s [127]. Two of them, CHIP and UBE4B are able to mediate ubiquitination and degradation of p53.

CHIP (carboxyl terminus of HSP70-interacting protein) is a protein that is highly expressed in adult striated muscle and in most cells in culture. It associates with the chaperones Hsc70 and Hsp70 and inhibits their activity [128]. Most interestingly, CHIP not only mediates poly-ubiquitination and degradation of wild-type p53 but also of mutated p53 [89]. Ubiquitination of wild-type and mutated p53 by CHIP was further enhanced by the presence of Hsc70 [90]. CHIP seems not only to degrade proteins that associate with chaperones, it also appears to be particularly required for the degradation of proteins during senescence, a permanent cell cycle arrest [129,130]. The p53 protein is also highly linked to senescence as activation of p53 can lead to cell cycle arrest, apoptosis and senescence [131]. Interestingly, p53 levels are high under pre-senescent conditions and decrease when cells reach senescence while the protein levels of CHIP show the opposite characteristic and are high in “late passage cells” [91]. This behavior is clearly distinct to many other E3s for p53 including MDM2, COP2, PIRH2 or TOPORS that are either reduced with increasing age or not altered. CHIP is not only induced with aging, it also changes from an entirely cytoplasmic localization in young cells to a partly cytoplasmic/partly nuclear localization in senescent cells [91]. These observations strongly point to a specific role of CHIP-mediated degradation of p53 in old and senescent cells, a hypothesis that is further supported by the observation that downregulation of e.g., MDM2 leads to high accumulation of p53 both in early and late passage cells while downregulation of CHIP stabilizes p53 only in late passage cells [91]

UBE4B, a mammalian homolog of the UFD2 protein of *S. cerevisiae,* is not only an E3 but also an E4, a protein that is in some cases required to ensure the proper formation of the ubiquitin chain [132]. UBE4B is especially expressed in neuronal tissue and its genetic deletion leads to early embryonic death due to extensive apoptosis. Heterozygote mice survive but develop a neurological disorder [133]. p53 can be ubiquitinated and degraded by UBE4B alone. However, UBE4B also interacts with MDM2 and the two proteins can form a ternary complex with p53 which boosts p53 polyubiquitination and degradation tremendously in comparison to ubiquitination and degradation of the tumor suppressor protein in the presence of the single E3 [134]. Interestingly, while p53 was observed to be primarily monoubiquitinated or multi-monoubiquitinated by MDM2 in the absence of UBE4B, this was shifted to polyubiquitination in the presence of MDM2 and UBE4B. The increased polyubiquitination of p53 under these conditions was accompanied by a decrease in p53 levels and a decrease in transcription of the p53 target genes p21 and MDM2 [134]. Similar to MDM2-mediated degradation of p53, UBE4B also enhanced p53 degradation mediated by PIRH2 and COP1 [92]. Surprisingly, a mutated form of UBE4B that was unable to mediate p53 degradation was still able to reduce p53-dependent transactivation, suggesting that UBE4B may use several ways to control p53 activity [134]. UBE4B is a target gene of p53 and this negative feed-back loop ensures low p53 levels [93]. As UBE4B also binds to p53 phosphorylated at serine 15 and promotes its degradation, it is most likely also involved in the shut-off of the DNA damage response [93].

#### 4.1.3. E3s That Require Complex Formation

While most enzymes modify p53 as single enzymes or dimers, some E3s are higher order complexes. The best characterized mammalian multi-subunit E3 is the SCF complex, a complex composed of 4 subunits, Skp1, Cul1/Cdc53, Roc1/Rbx1/Hrt1 and an F-box protein. The F-box protein is responsible for substrate recognition and binding while the entire complex provides the E3 activity [135]. One of these F-box proteins is FBXO42, also known as JFK (Jinfukang). FBXO42 is expressed in most human tissues with highest expression in heart and skeletal muscle [136]. FBXO42 is part of an SCF complex and it requires this association to destabilize p53 [136]. The FBXO42-containing SCF-complex inhibits p53-dependent transcription, and FBXO42 depletion stabilizes p53, promotes apoptosis, induces cell cycle arrest and sensitizes cells to ionizing radiation-induced cell death [136]. Like most other F-box proteins, FBXO42 requires phosphorylation of its substrate for full recognition [137]. Therefore, p53 is only recognized as a substrate of FBXO42 upon prior phosphorylation in its central domain, a modification that is implemented by CSN5, a COP9 signalosome-associated kinase. Inhibition or knockdown of COP9 or CSN5 impairs FBXO42-promoted p53 degradation resulting in enhanced p53-dependent transcription, growth suppression, cell cycle arrest and apoptosis [137]. Like most other E3s for p53, FBXO42 is transcriptionally activated by p53 and thus forms an auto-regulatory negative feedback loop with p53 [137].

### 4.2. E3s That Mediate p53 Ubiquitination without Promoting Degradation

**Figure 5:**
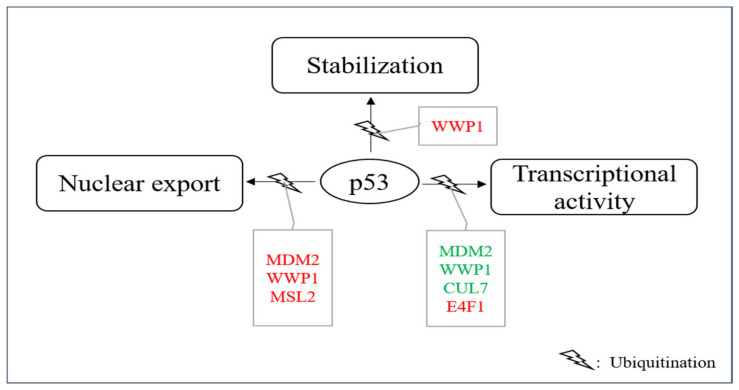
E3s that modify p53 without targeting it for degradation. A group of E3s including MDM2, MSL2, WWP1, CUL7, UBC13 and E4F1 decorate p53 with ubiquitin leading to nuclear export, enhanced stability and/or enhanced or reduced transcriptional stability. Ligases colored in red increase the individual activity of p53 and ligases shown in green reduce it.

In addition to proteasome-mediated degradation, ubiquitination of p53 can also have other consequences. For instance, it has been described that ubiquitination can promote nuclear export, stabilize the p53 protein or affect its transcriptional activity even without modulating its abundance (Table 2, Figure 5). Occasionally, the same E3 can even do both, promote p53 ubiquitination and its degradation and promote ubiquitination connected to other consequences. These sometimes even opposing activities may depend on the level of the E3, on different ubiquitin-linkages, on post-translational modifications of the E3, on the presence of other enzymes and on co-factors or on the cellular context in general.

The most prominent E3 that mediates p53 ubiquitination for other purposes than degradation is MDM2. MDM2 is able to ubiquitinates six C-terminal lysine residues of p53 [59]. While high levels of MDM2 or MDM2 together with an appropriate E4 mediate p53 polyubiquitination and subsequent degradation, low levels of MDM2 promote monoubiquitination of p53 and this monoubiquitinated p53 accumulates in the cytoplasm. p53 was also cytoplasmic when it was monoubiquitinated by genetic engineering, demonstrating that the modification of p53 is essential for its subcellular localization and not a putative shuttle function of MDM2 [138]. Ubiquitination of the six C-terminal lysine residues is essential for this process as ubiquitination at these sites leads to the exposure of a nuclear export signal of p53 in the C-terminal part of the protein [139]. According to its increased cytoplasmic localization, the transcriptional activity of p53 is reduced when it is monoubiquitinated. As a fraction of the cellular p53 protein typically localizes to the cytoplasm even under normal growth conditions, it is possible that this share of the p53 protein is normally cytoplasmic because of its monoubiquitination due to the usually low levels of MDM2 in unstressed cells [146,147]. This storage of p53 in the cytoplasm could serve as a rapid and reversable mean for downregulating p53 function. Alternatively, it could provide a mechanism for shuttling p53 to the cytoplasm where it can execute transcription-independent activities (e.g., activation of the mitochondrial cell death program) when needed.

MSL2 (male-specific lethal 2) is a nuclear, RING-containing protein of the MSL complex [148]. MSL2 was found to be associated with endogenous p53 after overexpression of the E3 in U2OS cells. MSL2 ubiquitinated p53 at lysine residues K351 and K357 in a RING-dependent manner [140]. After ubiquitination of these lysine residues, p53 accumulated in the cytoplasm (Figure 5). The cytoplasmic translocation was prevented by the drug Leptomycin B, demonstrating an involvement of the nuclear export machinery in the translocation process. Ubiquitination of p53 by MSL2 also occurred in MDM2-negative cells and is thus independent of MDM2 [140]. Although the lysine residues that are ubiquitinated by MSL2 are different from the ones that are ubiquitinated by MDM2, they might contribute to the same mechanisms which is alteration of p53 conformation and presentation of the nuclear export signal of p53 to the cellular export machinery. DNA damage leads to a rapid increase in MSL2 levels which occurs already at 30 min after ionizing irradiation and appears to be independent of p53 as there was no increase in MSL2 transcription [149]. This increase in MSL2 protein after DNA damage could contribute to an increase also of transcription-independent activities of p53 during the DNA damage response.

Also, WWP1 (WW domain-containing E3 protein 1), an E3 that is particularly found at cellular endosomes, mediates p53 nuclear export and suppresses its transcriptional activity. WWP1 is a member of the NEDD4 subfamily of E3s [150]. Like the other members of this family, WWP1 contains an N-terminal C2 domain, four tandem WW domains and a C-terminal catalytic HECT-domain. The C2 domain is a structural domain that is often found in proteins that bind phospholipids and target proteins to cell membranes. The four WW-domains mediate protein-protein interactions and bind particularly well to proline-rich polypeptides [151]. The interaction of p53 and WWP1 has been found by searching for ubiquitin ligases that associate with the proline-rich domain of p53. Later it turned out that not the proline-rich region, but the DNA binding domain, is the primary site of interaction of p53 with the E3. Nevertheless, the proline-rich domain is important for this interaction as its deletion weakens the interaction of p53 with WWP1. Although all members of the WWP family possess the proline-binding WW motif, WWP1 and to a lesser extend WWP2 are the only family members that associate with p53 [141,151]. WWP1 associates with p53 and ubiquitinates the tumor suppressor protein in a HECT-domain-dependent manner, however, without targeting it for degradation. Instead, the p53 protein becomes even more stable. Ubiquitination of p53 by WWP1 is, however, modest in comparison to ubiquitination of p53 by MDM2. WWP1-mediated stabilization of p53 is associated with an increase in cytoplasmic p53 and decreased transcription of p53 target genes (Figure 5). Knocking-down WWP1 in hepatoma carcinoma cells resulted in reduced growth and increased apoptosis in the tumor cells [141,142]. Ubiquitination of p53 by WWP1 is independent of MDM2 as it is also seen in in MDM2-knock-out cells. WWP1 is induced after DNA damage at the mRNA level, however, while p53 stimulates expression of most of the E3s that modify it, it reduces WWP1 expression and may thus not be involved in its induction after DNA damage (Chen et al.). [141,152].

Another E3 that modifies p53 without targeting it for degradation is CUL7 (Cullin7) [143]. CUL7 is a member of the Cullin protein family, a family of evolutionarily conserved proteins that bind to the RING protein ROC1 (also known as RBX1, HRT1 or Sag1), thus constituting a functional E3 [153]. The N-terminal region of the Cullin is responsible for substrate recruitment while the C-terminal part interacts with ROC1. CUL7 is localized in the cytoplasm and binds directly to p53 [143]. However, CUL7 neither affects p53 stability nor its subcellular localization, probably because it only mediates mono- or diubiquitination of p53 that is not sufficient for targeting p53 for degradation (Figure 5) [144]. Surprisingly, deletion of the ROC1-binding sequence did not alter CUL7-mediated p53 ubiquitination, implying that ROC1 is not essential for this process [144]. Whether CUL7 associates with another E3 for ubiquitinating p53 remains to be determined. Co-expression of CUL7 reduced p53-mediated transactivation and constitutive ectopic expression of CUL7 increased cell proliferation and delayed an UV-induced G2 arrest in U2OS cells but not in H1299 cells that do not express p53. Also, deletion of the N-terminal domain of CUL7 or a mutation that disrupted p53 binding abolished the ability of CUL7 to enhance U2OS cell proliferation, indicating that this effect of CUL7 depends on p53 [144]. CUL7 is induced after DNA damage at the protein and RNA level, which seems to be independent of p53 as it was also induced in p53-negative HCT116 cells and with the same kinetics as in the corresponding wild-type cells [154].

E4F1 was first identified as a cellular target of the viral oncoprotein E1A. Later it was found that it possesses E3 activity towards itself and that it binds to p53 and induces p53-dependent cell cycle arrest [145,155]. These evidences raised the intriguing possibility that E4F1 might also ubiquitinate p53 and regulate its activities. Indeed, E4F1 stimulated oligo-ubiquitylation of p53 in the hinge region of p53 that contains a cluster of three lysine residues (K319, K320, K321). Mutation of these sites rendered p53 insensitive towards E4F1-mediated ubiquitination [145]. However, despite equipping p53 with K48-linked ubiquitin chains, E4F-1-mediated ubiquitination did not increase p53 degradation. Instead, E4F1 overexpression induced a moderate, but reproducible, stabilization of p53 [145]. This E4F1-modified p53 was predominantly found at the chromatin where it stimulated transcription of growth arrest inducing genes while p53-dependent anti-apoptotic activities were suppressed (Figure 5) [145].

### 4.3. E3s That Mediate Neddylation and Sumoylation of p53

**Figure 6:**
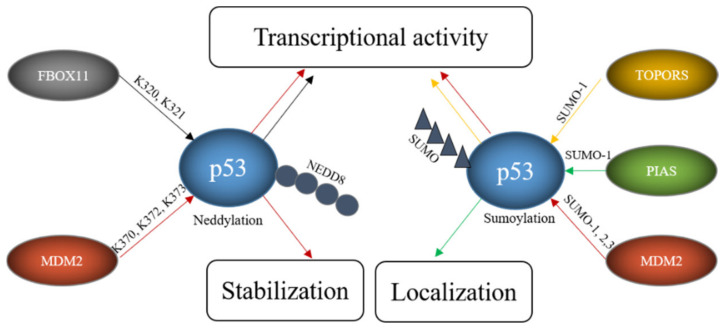
E3s modify p53 with SUMO and NEDD8. FBOX11 modifies p53 with NEDD8 and E3s like TOPORS or proteins of the PIAS family modify p53 with SUMO while MDM2 can modify p53 both with SUMO and NEDD8. These modifications alter p53 stability, localization and transcriptional activity.

In addition to ubiquitination, p53 can also be modified with NEDD8 (neural precursor cell expressed, developmentally downregulated 8) and SUMO (small ubiquitin-like modifier) [156,157,158]. In contrast to ubiquitination and neddylation, where there is only one protein in cells that can be attached to a target protein (ubiquitin and NEDD8 respectively), four SUMO proteins are expressed in cells. SUMO1, SUMO2 and SUMO3 are widely expressed while the expression of SUMO-4 is limited to certain organs [159]. SUMO4 furthermore contains a proline residue close to the diglycine motif, which may prevent its processing [160]. Since NEDD8 or SUMO compete with ubiquitin for the same lysine, these modifications frequently lead to stabilization of the target protein. Like ubiquitination, the sumoylation and neddylation processes include three steps. Prior to neddylation of a target protein, the NEDD8 precursor protein is activated by the NEDD8-specific E1, a heterodimer consisting of APP-BP1 (amyloid precursor protein binding protein 1) and UBA3 (ubiquitin-like modifier activating protein 3) in an ATP-dependent manner. The activated NEDD8 is loaded onto the NEDD8-specific E2 UBC12. Finally, a NEDD8-specific E3 conjugates NEDD8 to the target protein. Unlike ubiquitin, NEDD8 does not form chains [161]. The process of sumoylation follows the same pattern. To start the reaction, the pro-peptide of one of the SUMO proteins is processed to expose its C-terminal diglycine motif. This is usually performed by one of several SENPs (Sentrin/SUMO-specific protease). The processed SUMO is then activated in an ATP-dependent manner by a SUMO-activating enzyme, a heterodimer of SAE1 (SUMO-activating enzyme 1) and SAE2. In the next step, the SUMO protein is loaded onto the SUMO conjugating enzyme UBC9 (ubiquitin-conjugating enzyme 9). Finally, a SUMO E3 catalyzes the covalent attachment of SUMO to the target protein. In contrast to ubiquitination, sumoylation only occurs when the target protein possesses a sumoylation consensus motif consisting of ψ-K-X-E/D where ψ is any hydrophobic amino acid, K is the lysine to be sumoylated, E is glutamic acid and D is aspartic acid [162]. Several ligases can modify p53 with NEDD8 and/or SUMO including MDM2, PIAS proteins, FBXO11, and TOPORS (Table 3, Figure 6).

MDM2 can actually decorate p53 with ubiquitin, as described above, as well as with NEDD8 and SUMO. Neddylation of p53 requires a direct interaction of p53 with MDM2. It occurs at the C-terminal lysine residues K370, K372 and K373 and is accompanied by a decrease in p53 activity [158]. Sumoylation of p53 by MDM2 employs SUMO 1 and SUMO2/3 and occurs at lysine K386 within a SUMO consensus motif. Sumoylation even occurs to the conformational mutants p53^R273H^ and p53^R175H^. As murine p53 lacks the consensus motif, this modification is not seen in mice [157]. While ubiquitination of p53 by MDM2 can be reconstituted in vitro, this was not possible for sumoylation, implying that a particular subcellular location or additional proteins are required [157]. Nevertheless, binding of p53 to MDM2 is required for sumoylation as shown by a p53 mutant (p53^14Q, 19S^) that cannot bind to MDM2 and that is poorly sumoylated [169]. The sumoylation activity of MDM2 is independent of the RING domain of MDM2 and stimulated by the presence of p14/p19^ARF^, the product of the alternative reading frame of the INK4a/b locus [170]. The nucleolar protein p14/p19^ARF^ sequesters MDM2 in the nucleolus and the targeting of MDM2 and p53 to the nucleolus seems to be important for MDM2-mediated sumoylation of p53 [42]. Despite the importance of the nucleolar localization, an engineered p53 protein that was directly targeted to the nucleolus showed only modest sumoylation. Co-transfection with p14/p19^ARF^, however, led to a strong increase in p53 sumoylation, suggesting that nucleolar targeting, the presence of MDM2 and p14/p19^ARF^ are all required for efficient sumoylation of p53 [169]. Mdm2-mediated sumoylation of p53 is also promoted by the ribosomal protein L11 [157]. While there is large consent that p53 is sumoylated by MDM2, the reported functional consequences are contradictory. Some researchers observed a modest increase in p53 activity after co-expression of p53, SUMO1 and UBC9 and a slightly compromised pro-apoptotic activity after mutation of the SUMO consensus motif of p53 while such an increase in p53 activity was not seen by others [163,171]. Interestingly, p53 modified by SUMO2/3 was more efficient in activating the synthetic PG13-promoter and the p21-promoter but not the BAX-promoter suggesting that sumoylation may regulate transcription of only a subset of target genes [157]. This promoter-dependent behavior of p53 modified with SUMO2/3 could eventually provide an explanation for the contradictory results that have been obtained with p53 modified with SUMO1. A similar debate is ongoing about the effect of sumoylation of p53 on its subnuclear localization. While Carter and Vousden observed at least a partial increase in nuclear export of p53 that was sumoylated with SUMO1, Kwek and co-workers found sumoylated p53 tightly bound to chromatin in the cell nucleus [171,172]. Interestingly, modification of p53 with SUMO2/3 was increased after treatment of cells with H_2_O_2_ and overexpression of SUMO2/3 promoted a premature senescence phenotype that was reduced by downregulating p53. These results suggest that modification of p53 with SUMO2/3 plays a role in the oxidative stress response and in the induction of senescence [173]. As MDM2 is also an important E3 for p53 ubiquitination and also mediates p53-neddylation, this raises the question how MDM2 can change from a ubiquitin-specific E3 to an E3 for NEDD8 or SUMO. At least part of this promiscuous behavior of MDM2 lies in the decoration of MDM2 itself with post-translational modifications. Phosphorylation of MDM2 at tyrosine Y281 and Y302 by the SRC kinase leads to the recruitment of the NEDD8-specific E2 UBC12 and by this converts MDM2 from an E3 for ubiquitin to an E3 for NEDD8 [174].

Like MDM2, TOPORS not only modifies p53 with ubiquitin, but also with SUMO1. This modification is direct as it was reproducible in an in vitro system. While the RING domain of TOPORS was obligatory for p53 ubiquitination, it was optional for p53 sumoylation [164]. Overexpression of TOPORS in Hela cells stimulated p53 sumoylation and was accompanied by an increase in p53 protein level and activity. Like in the case of MDM2, the preference of the E3 for SUMO1 or ubiquitin was regulated by post-translational modifications of the E3. While phosphorylation of TOPORS by the Polo-like kinase 1 (Plk1) enhanced p53 ubiquitination and degradation, it reduced TOPORS-mediated sumoylation of p53 [67].

A major family of E3s for p53 sumoylation is the PIAS family. All members of this family, PIAS 1, PIAS2, also known as PIASx, PIAS3 and PIAS4, also known as PIASγ, interact with p53, stimulate SUMO conjugation to p53 in a reconstituted in vitro system and increase the modification upon ectopic expression in vivo [165,166]. However, whether all family members sumoylate p53 also at physiological expression levels remains to be determined. Nevertheless, for PIAS4 it has been shown that the amount of sumoylated p53 is reduced in HEK293 cells upon its RNAi-mediated depletion [167]. As some PIAS proteins are expressed in a tissue-specific manner, it is most likely that different PIAS proteins sumoylate p53 in distinct tissues. Not all PIAS proteins have been studied in detail for activities towards p53, but for PIAS4 it has been shown that its overexpression correlates with activation of p53 target genes and induction of cellular senescence in primary human fibroblasts [167]. Since these effects depend on the ligase activity of PIAS4, it is tempting to conclude that they are a direct consequence of p53 sumoylation, although this has not been formally demonstrated. Earlier data also showed suppression of p53-mediated transcription of a p21-reporter and of endogenous p21 in the presence of PIAS4. Also, for PIAS1, it has been described that it can both, activate and repress p53-driven transcription [165,166,175]. Eventually the SUMO system may convey promoter-specific effects, an idea that is supported by the observation that PIAS4 affected p53-mediated expression of p21 but did not compromise BAX expression [176]. Thus, PIAS family members may be involved in differential regulation of selected p53 target genes.

FBXO11, a member of the F-box protein family and a component of the SCF ubiquitin ligase complex, was also found to be a NEDD8-E3 for p53. Although F-box proteins frequently mediate ubiquitination and degradation of their target proteins, this is not the case for SCF-complexes containing FBXO11 and p53. Instead, FBXO11-containing SCF-complexes attached NEDD8 to p53 involving the lysine residues K320 and K321. This modification inhibited p53 activity without altering its stability or subcellular localization [168].

### 4.4. E3s That Act. on p53 without Modifying It

While most E3s control p53 abundance and/or activity by directly decorating the tumor suppressor protein with ubiquitin or the related ubiquitin-like small modifiers, there are also some proteins with E3 activity that regulate p53 abundance and activity by a different mode. One of these proteins is TRIM25. TRIM25 is an estrogen-inducible member of the TRIM-family. As such, it has a RING domain and its ubiquitin ligase activity has been demonstrated by showing that it ubiquitinates and degrades 14-3-3σ [177]. However, although TRIM25 physically interacts with p53, it does not mediate p53 ubiquitination nor its modification with other ubiquitin-like small modifiers. Instead, TRIM25 leads to p53 stabilization by reducing the interaction of MDM2 with p300, an E4 enzyme required for the formation of polyubiquitin chains on p53 [178]. At the same time, TRIM25 inhibits the transcriptional activity of p53 by reducing the abundance of p300 and thus p53 acetylation [178].

PARC (p53-associated, Parkin-like cytoplasmic protein), also known as CUL9, an RBR-type E3, was identified during a search for cytoplasmic binding partners for p53 [179]. RBR-type E3s have more recently been categorized as a new family of E3s. They contain three highly conserved domains: Ring1, IBR (InBetweenRING) and RING2 and share common features with the RING- and the HECT- domain ligases [51]. PARC associates with p53 and the majority of cytoplasmic p53 appears to be associated with PARC. However, PARC did not modify p53 [179]. Downregulation of PARC resulted in enhanced nuclear localization of p53 and upregulation of p53-dependent apoptosis while overexpression of PARC resulted in enhanced cytoplasmic sequestration of p53 [179]. Deletion of PARC enhanced DNA damage, led to spontaneous tumor development and accelerated Eμ-Myc-induced lymphomagenesis [180,181]. In SK-N-AS neuroblastoma cells, which respond badly to the genotoxic drug etoposide, enhanced deletion of PARC the response to DNA damage [179]. As PARC retains p53 in the cytoplasm, expression of the p53 target gene p21 was significantly reduced, both under normal conditions and in response to DNA damage [181].

The PML (promyelocytic leukemia) protein, a tumor suppressor protein that is frequently implicated in leukemia, is best known for its translocation with the retinoic acid receptor alpha (RARa) resulting in the PML-RARa protein, and for the organization of PML nuclear bodies [182,183]. PML physically interacts with p53 and enhances p53′s proapoptotic and transcriptional activity [184]. Cells lacking PML showed a reduced propensity to undergo senescence or apoptosis in response to p53 activation despite the induction of several p53 target genes [185]. PML comes in several isoforms and at least one of them (PML III) targets p53 into nuclear bodies [184]. This colocalization of p53 and PML is enhanced in response to DNA damage [184]. PML is a p53 target gene and p53 stimulates PML induction in response to oncogenes and after DNA damage [185].

## 5. Tumor Virus-Associated E3s

Most viral infections of humans or animals do not cause cancer. This is due to the fact that only a few viruses are able to cause cancer and most infections even with a tumor virus remain asymptomatic. However, about 16% of human cancers are estimated to be related to viral infections [186]. Most of these cancers are caused by human papilloma viruses, hepatitis B and C viruses, the Epstein-Barr virus, the human immunodeficiency virus or the human herpes virus [186,187].

As p53 is an important tumor suppressor protein, it is not surprising that tumor viruses aim to inactivate p53 and thus encode E3s or E3-associated proteins. The first viral protein that was found to target p53 for proteasomal degradation was the E6 protein [188]. The E6 protein is encoded by the human papillomaviruses (HPV) types 16 and 18 and is one of two HPV-proteins that are expressed in HPV-associated cancers [189]. The E6 protein requires the cellular protein UBE3A for binding to p53. It forms a stable complex with UBE3A and the two proteins then form a ternary complex with p53. This complex allows the ubiquitination of p53 at multiple lysine residues after HPV infection resulting in rapid degradation of p53 [83]. Only oncogenic HPVs target p53 for degradation but not the benign ones [190].

Another viral protein that targets p53 for degradation is the adenoviral protein E1B55K. Adenoviruses are medium-sized DNA viruses that were initially isolated from human adenoids [191]. Today more than 50 distinct adenoviral serotypes are known. E1B55K is one of two genes that are transcribed from the E1B locus. Both genes are needed to block apoptosis upon adenoviral infection that is induced by the E1A gene [192,193]. E1B55K blocks p53 activity in two ways. It binds to p53 and turns it from an activator to a repressor of gene transcription and it associates with the viral protein E4orf6 to promote p53 degradation [194,195]. E1B55K and E4orf6 interact directly with p53, mediate p53 ubiquitination, target p53 for degradation, block p53-dependent apoptosis, and accelerate tumor formation. However, additional proteins are required for full activity [196,197]. Affinity purification of E4orf6-containing complexes with subsequent mass spectrometry showed that E4orf6 associates with CUL5, elongin B and elongin C of the Cullin complex and transfection of elongin B, elongin C, CUL5 and Rbx1 of the Cullin complex together with E1B55K and E4orf6 resulted in efficient ubiquitination and degradation of co-transfected p53 [197]. However, E1B55K not only mediates the decoration of p53 with ubiquitin, Muller and Dobner observed a higher molecular weight band of p53 in adenovirus-transformed HEK293 and HEK293T cells and identified this higher molecular weight band as p53 modified with SUMO2/3 [198]. The sumoylation of p53 by E1B55K occurred at lysine K386 within the SUMO consensus site and required binding of E1B55K to p53 and nuclear localization of the ligase. While Muller and Dobner observed p53-sumoylation with SUMO2/3, Pennella et al., found that E1B55K decorates p53 with SUMO1 and that this modification occurs during adenoviral infection, inhibits p53 transcriptional activity and mediates its nuclear export [198,199]. Modification of p53 with SUMO1-was reconstituted *in vitro*, proving that this is a direct activity of E1B55K [199]. Like many other sumoylated proteins, sumoylated p53 is highly abundant in PML (promyelocytic leukemia) bodies, a membrane-less subnuclear structure mainly organized by the PML protein [183,200]. Co-expression of E1B-55K together with p53 leads to colocalization of p53, E1B-55K and the PML protein within PML-bodies and to the colocalization of p53 and E1B-55K in cytoplasmic inclusion bodies [198]. But the E1B locus is not the only adenoviral gene locus that mediates p53 modification. Infection of cells with adenoviruses leads to a strong E1A-mediated induction of E4F1, an E3 for p53 [145,201]. E4F1-mediates oligo-ubiquitylation in the hinge region of p53 and stimulates a p53-dependent transcriptional program that specifically controls the cell cycle [145].

Another viral protein that mediates p53 modification without affecting its degradation is ICP0, the product of an immediate early gene of the human herpes simplex virus type 1 (HSV-1), a human pathogen that causes oral and genital herpes. ICP0 interacts with various cellular proteins, including Cyclin D3, Elongation factor EF-1δ, and with the ubiquitin-specific protease USP7 (also known as HAUSP). Virus mutants that do not express ICP0 are severely impaired in their ability to replicate. A crucial feature of ICP0 activity is its RING domain that equips ICP0 with E3 activity. ICP0 interacts directly with p53 and mediates p53 ubiquitination [202]. However, p53 levels were not greatly affected by a HSV-1 infection and the regulation of the cell cycle by HSV1 occurred independently of p53 [202]. p53 was, however, recruited to compartments of viral DNA replication and co-localized there with ICP0 [203]. This co-localization was most prominent after overnight infection when the majority of cells had entered a non-productive infection state. Following infection with HPV1, ICP0 strongly reduced UV-mediated induction of apoptosis, indicating that despite the marginal impact on p53 levels, HPV1 may affect p53 activities under certain conditions [203].

In summary, by mediating ubiquitination and sumoylation resulting in p53 degradation, its nuclear export or by shifting its activity towards cell cycle arrest instead of apoptosis, different tumor viruses have found different ways to change p53 activity according to their needs. However, not all tumor viruses use the ubiquitin-proteasome system to inactivate p53. Binding to the transactivation domain of p53 (LargeT antigen of the SV40 virus), expression of anti-apoptotic proteins (LMP1 of Epstein-Barr virus) and promotion of p53 mutations (Hepatitis B virus) are also used by tumor viruses to inhibit p53 activity [204,205,206].

## 6. E3s Targeting Mutated p53

While E3s targeting wild-type p53 have been widely investigated in the last decades, only little is known about E3s targeting mutated p53. About 50% of human cancers possess mutated p53. Mutated p53 has a largely extended half-life, activates a different set of genes and has a high tendency to aggregate [207,208]. Most cancer-related mutations are single amino acid substitutions within the DNA binding domain that reduce the contact with p53-responsive elements in promoters of target genes, the so called “contact mutants”, or that change the three-dimensional structure of p53, the so called “conformation mutants” [209]. Although mutated p53 is very stable, at some point it is turned-over. The proteasome is also an important structure for the degradation of mutated p53, although also chaperone-mediated autophagy is used [210]. Since wild-type and mutated p53 usually differ only in one amino acid, one would expect that a similar cohort of E3s would also modify mutated p53. However, from the large group of E3s descried above, only MDM2, CHIP, PIRH2, COP1 and TRIM71 have been reported to target mutated p53 for degradation, although ARF-BP1, CUL7 and CUL9 also have been shown to associate with mutated p53 [88,211,212,213].

MDM2 and CHIP are certainly the most investigated E3s for mutated p53. Interestingly, while MDM2 targets wild-type p53 for rapid proteasomal degradation, mutated p53 is much more stable although both bind to MDM2 [214]. However, while the interaction of MDM2 with wild-type p53 mainly occurs through the N-terminal interaction site with only minor contribution of a central and C-terminal interaction site, the interaction of MDM2 with mutated p53 occurs mainly via a C-terminal interaction site. This altered interaction with MDM2 resulted in a strongly reduced activity of MDM2 [211]. Differing information exists whether mutated p53 is ubiquitinated at all. While Lukashuk and Vousden observed ubiquitination of mutated p53 that resulted in cytoplasmic translocation, this could not be confirmed by Li and co-workers, who observed only non-ubiquitinated mutated p53 in cells that was predominantly nuclear [211,215]. The main obstacle, why MDM2 or CHIP, although principally able to ubiquitinate mutated p53 and to mediate its degradation, show only limited activity towards mutated p53 is that mutated p53 is complexed to the HSP90 (heat shock protein 90) machinery. This association with HSP90 and HDAC6 prevents on the one hand the aggregation of mutated p53, but also interferes with its accessibility for E3s. Wild-type p53 is unable to form stable complexes with HSP90 and is therefore not stabilized in tumor cells. Most interestingly, in non-transformed cells, MDM2 is able to target mutated p53 for degradation, suggesting that it is rather the cellular environment than the E3 itself that is altered [215]. The reason for this is most likely that HSP90 is highly upregulated in cancer cells. Downregulation of HSP90 or its pharmacological inhibition liberates p53 from the interaction with HSP90 and allows its ubiquitination and degradation [215]. Nevertheless, there is also evidence that CHIP is able to target aggregating mutated p53. Maan and Pati observed increased levels of aggregating mutated p53 after downregulation of CHIP and mapped the binding of aggregated p53 to the U-box domain of CHIP. CHIP decorated the mutated p53 with lysine K63-linked polyubiquitin chains and targeted it for degradation by autophagy [89].

Although MDM2 and CHIP are certainly the most investigated E3s for mutated p53, a few others have also been described to bind to mutated p53, ubiquitinate it and target it for degradation. Treatment of cells with arsenic trioxide decreased mutated p53 levels in a dose-dependent manner through the proteasome pathway [216]. Arsenic trioxide induces expression of the E3 PIRH2. Overexpression of PIRH2 decreased p53 levels in HaCaT and PaCa-2 cells in a RING-dependent manner while downregulation of PIRH2 increased p53 abundance and reduced proliferation. PIRH2, furthermore, co-precipitated with p53 and ubiquitinated p53 in vitro [212]. TRIM71 was also found to bind to the N-terminal domain of mutated p53 [213]. TRIM71 decorated mutated p53 with K11-, K27-, K29- and K63-linked polyubiquitin chains and targeted it for proteasomal degradation in an MDM2-independent manner while downregulation of TRIM71 stabilized mutated p53 protein. Stable overexpression of TRIM71 in ES-2 and OVCA420 cells reduced the expression of target genes of mutated p53 including c-Myc, MMP3 or RANGAP1 and suppressed cell proliferation, invasion and tumor growth in xenografts while it barely affected expression of wild-type p53 and proliferation or invasion of ovarian cancer cells with wild-type p53 [213]. TRIM71 is frequently under-expressed in human cervix, esophagus, lung and kidney cancer, but no significant prognostic clinical value could be attributed to TRIM71 [213].

Another E3 for mutated p53 is RNF128. RNF128 has two isoforms. While the isoform Iso1 showed only limited ubiquitin ligase activity for p53, the isoform Iso2 potently reduced levels of mutated p53 in Barett’s esophagus cells [217].

Also, viral proteins are able to target mutated p53 for degradation. Mutated p53 co-precipitated with human papillomavirus E6/UBE3A, however, this interaction depended strongly on the type of the p53 mutation [218]. All mutated p53 proteins that were recognized by the conformation-specific anti-p53 antibody Pab1620 associated with E6/UBE3A, implying the need for an overall wild-type structure for being targeted [219]. Mutated p53 that associated with E6/UBE3A was also ubiquitinated and degraded by the E3.

Thus, although most of the mutated p53 is locked in the HSP90 complex and thus largely inaccessible for E3s, leading to its high stability, several E3s have been shown to also show activity towards mutated p53.

## 7. E3s Targeting p53 Isoforms

Due to alternative initiation sites at codon 40 and codon 133, an internal promoter in intron 4 and alternative splicing sites, a total of 12 different isoforms (p53, p53β, p53γ, Δ133p53α, Δ133p53β, Δ133p53γ, Δ160p53α, Δ160p53β, Δ160p53γ, Δ40p53, Δ40p53β and Δ40p53γ) are transcribed from the *TP53* gene [220]. These isoforms are expressed to varying degrees in different normal cells and tumor tissues [220,221]. Breast cancer patients expressing mutated p53 together with the p53γ isoform have been found to have a much better prognosis than patients expressing only mutant p53, without p53γ, showing that the expression of these isoforms are also relevant for carcinogenesis [222].

**Figure 7:**
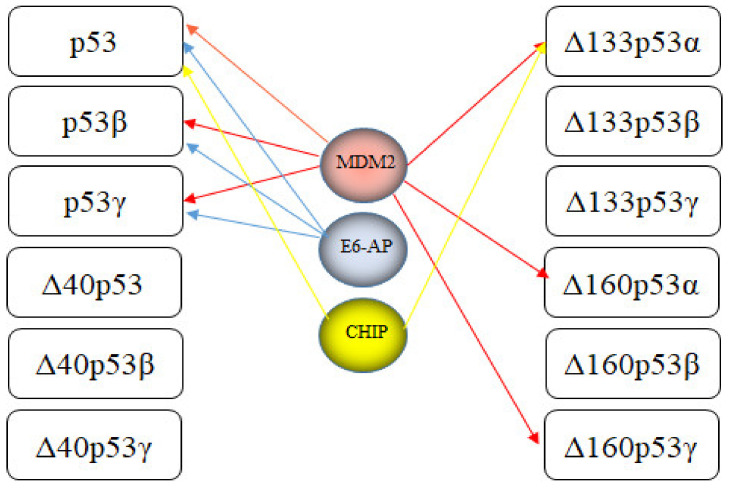
E3s that modify p53 isoforms. The p53 protein comes in twelve isoforms. Only for some of them, E3s have been identified. MDM2, E6AP and CHIP modify some of the p53 variants.

Several isoforms of p53 show tissue specific expression, implying a selective regulation [223]. However, despite their identification more than fifteen years ago, our knowledge about the targeting of p53 variants by E3s is limited. However, for some isoforms, data are available (Figure 7). Treatment with the proteasome inhibitor MG132 increased the abundance of p53β, indicating that it is degraded by proteasomes, and overexpression of MDM2 promoted its degradation in a dose-dependent manner. Mdm2 also modified p53β with NEDD8 and this modification increased p53β stability, probably by preventing its ubiquitination [222]. Several other isoforms of p53 have also been analyzed and showed a large variety of half-lives after overexpression. While p53β was quite stable, Δ133p53γ and p53γ displayed half-lives of about 40 and 50 min, respectively and Δ133p53α and Δ133p53β showed half-lives between 110 and 135 min. The half-lives of Δ160p53 isoforms were in agreement with the degradation of their Δ133p53 counterparts [222]. Treatment of cells overexpressing p53 isoforms with MG132 greatly extended the half-lives of p53γ, Δ133p53α, Δ160p53α, Δ133p53γ and Δ160p53γ, while the stability of p53β, that had a relatively long half-life in the absence of an ectopic E3, was not affected. The isoforms Δ133p53β and Δ160p53β showed an intermediate response [222]. Ubiquitinated forms of all p53 isoforms accumulated in the presence of MG132, indicating that all are targets of E3s. Co-expression of Mdm2, however, only promoted degradation of p53β, suggesting that Mdm2 cannot modify other p53 variants [222]. The inability of Mdm2 to degrade Δ133p53 and its splice variants is not surprising, as these isoforms do not contain the N-terminal Mdm2 binding site. However, p53β and p53γ contain this binding site and co-immunoprecipitation experiments showed that they associate with Mdm2. Δ133p53 and Δ160p53 interacted with Mdm2 despite the absence of the N-terminal binding site, but at a very low level [222].

Δ133p53α has also been shown to associate under nutrient starvation with CHIP, together with proteins of the HSP90 family and other chaperones [224]. Downregulation of CHIP in early-passage MRC5 fibroblasts resulted in a significant reduction in Δ133p53α levels that was prevented when autophagy was inhibited, suggesting that Δ133p53α is also degraded by autophagy and that CHIP may have a protecting role [224].

Also, the E6/UBE3A complex degrades p53β and p53γ while Δ133p53α, Δ133p53β and Δ133p53γ were not targeted by E6/UBEA3 [222].

## 8. E3s and Tumorigenesis

p53 is a well-acknowledged tumor suppressor protein, which regulates biological processes that involve inappropriate cell expansion, tumor initiation and progression. p53 is frequently mutated in tumors and genome sequencing of thousands of tumors has confirmed that approximately half of all cancers harbor a p53 mutation [225]. The importance of p53 for tumor suppression has also been revealed experimentally. Mice, where the p53 gene was targeted to generate p53-knock-out mice developed largely normal, but they were prone to spontaneous development of neoplasms by an age of 6 month [226]. Furtherly, mice that have been manipulated to carry a mutated p53 gene (that is defective in initiating apoptosis but still able to induce cell cycle arrest (p53^R172P^, the murine equivalent to human p53^R175P^) suffer earlier from cancer than p53 wild-type mice but survive longer than p53-null mice [227]. Considering the important role of p53 for tumorigenesis, it is not surprising that several E3s that control p53 activity are involved in the modulation of tumorigenesis (Table 4). Most of these E3s promote p53 ubiquitination and degradation. Overexpression of these enzymes leads to a functional knock-out of p53 and thus promotes carcinogenesis. Overexpression of MDM2, for instance, has been observed in many types of cancers, particularly in cancers where p53 mutations are less frequent including breast cancer, prostate cancer, colorectal adenocarcinoma, esophageal squamous cell carcinoma, gastric cancer and non-small cell lung cancer, and high MDM2 levels are associated with poor prognosis of patients [228,229,230,231,232,233,234]. Like Mdm2, COP1 has been reported to be highly expressed in different human cancers. The increased expression of COP1 is associated with increased cell proliferation, cell transformation and tumor progression. Elevated COP1 levels were found in breast adenocarcinomas, ovarian adenocarcinomas, pancreatic cancer and hepatocellular carcinoma [235,236,237]. Downregulation of COP1 in hepatocellular carcinoma cells resulted in reduced proliferation and growth arrest and xenografts from these cells showed a dose-dependent decrease in tumor mass [235]. Yet, conflicting results have been reported for gastric cancer. While Li and coworkers observed overexpression of COP1 in gastric cancer and an association of high COP1 levels with poor survival, Sawada and coworkers reported downregulation of COP1 in gastric cancer [238,239]. As COP1 is an E3 for several oncogenes and tumor suppressor proteins including c-Jun, ETS, β-Catenin, STAT3, MTA1, p27, 14-3-3σ, and C/EBPα [237,240], it is possible that it may depend on the cellular context whether high levels of COP1 are beneficial or detrimental for tumor patients.

Also, the p53-E3 PIRH2 has been reported to be overexpressed in several cancers including hepatocellular carcinoma, head and neck cancer, lung cancer and prostate cancer [241,242,243,244]. However, while PIRH2 mediates p53 ubiquitination and degradation and thus has oncogenic activity, it also mediates poly-ubiquitination and proteolysis of the oncoprotein c-Myc [62,284]. PIRH2 mutant mice therefore display elevated levels of c-Myc and are predisposed for plasma cell hyperplasia and tumorigenesis. Moreover, low expression of PIRH2 in lung, ovarian and breast cancer correlated with lower survival times of cancer patients [284,285,286].

RNF1 is overexpressed in non-small cell lung cancer and hepatocellular carcinoma and is one of the biomarkers for a poor outcome of prostate cancer [71,245,246]. RNF1 depletion inhibited the proliferation and survival of p53 wild-type cancer cells by inducing cell cycle arrest and senescence, with only modest effects in p53-deficient cells [71]. However, RNF1 also activates the Wnt/β-Catenin signaling pathway and drives the transformation of hepatic progenitor cells into cancer stem cell-like cells in a p53-independent manner [287]. RNF1 also mediates ubiquitination of histone H2A which affects neuronal migration and axon guidance [288].

RNF2 is highly expressed in colon cancer, gastric cancer, B-cell lymphoma, Burkitt’s lymphoma and Hodgkin’s lymphomas [247]. Knockdown of RNF2 in p53-positive HCT116 cells resulted in significant more apoptosis than in p53-negative HCT116. However, RNF2 also mono-ubiquitinates H2A at lysine K199 at the promoter of the latent-transforming growth factor beta-binding protein 2 (LTBP2), resulting in the activation of TGF-β signaling and invasion of melanoma cells. RNF2 also induces the expression of Cyclin D2 and thus drives proliferation of melanoma cells [248].

TOPORS is highly expressed in normal human colon tissues, while TOPORS mRNA and protein levels are decreased in colon adenocarcinomas relative to normal colon, probably caused by increased methylation of a CpG island in the TOPORS promoter [249]. Overexpression of TOPORS inhibits cellular proliferation and induces cell cycle arrest and its absence leads to genetic instability and increased malignancy in mice, suggesting that TOPORS functions as a negative regulator of cell growth and as a DNA damage response and tumor suppressor protein [249,289].

Also, several E3s of the TRIM protein family are associated with cancer. TRIM24, for instance, is overexpressed in breast cancer, non-small cell lung cancer, head and neck squamous cell carcinoma, glioma, gastric cancer, bladder cancer and hepatocellular carcinoma [250,251,252,253,254,255,256]. Surprisingly, knocking-out TRIM24 also leads to hepatocellular carcinoma, suggesting that the right dose of the ligase is important for homeostasis [290]. The switch from a tumor suppressor protein to an oncogene may also rely on its interaction with and co-activation of nuclear receptors including the retinoid-X receptor, the retinoid-alpha receptor, the vitamin D3 receptor, the estrogen receptor or the progesterone receptor [291]. TRIM25 is reported to be upregulated in endometrial cancer, ovary cancer, prostate cancer, lung cancer and breast cancer and its overexpression is associated with poor prognosis, at least for breast cancer patients [257,258,259,260,261]. Downregulation of TRIM25 inhibits proliferation of breast cancer cells and tumor growth in vivo and migration and invasion of lung and gastric cancer cells [292,293,294]. TRIM28 is highly expressed in many epithelial cancers, including gastric cancer, ovarian cancer, glioma, hepatocellular carcinoma and breast cancer. Elevated levels of TRIM28 have been linked to poor prognosis and lower overall survival [262,263,264,265,266,295]. However, in the early stage of lung cancer, high expression of TRIM28 was also reported to be associated with a better overall survival [295] and mice with liver-specific ablation of TRIM28 showed an increase in hepatic adenoma, particularly in male animals [296]. Also, TRIM32 can act both, as an oncogene and as a tumor suppressor protein [297]. By mediating degradation of p53, over-expression of TRIM32 leads to decreased apoptosis, cell cycle arrest and senescence and to tumorigenesis in mice, and by mediating the degradation of the Abl-interactor 2, a protein that supports cell growth and motility, TRIM32 increases the aggressiveness of tumors [76,267]. As a tumor suppressor, TRIM32 mediates degradation of the anti-apoptotic protein XIAP which finally enhances the sensitivity towards anti-cancer drugs and of MYCN, a protein that controls asymmetric division of neuroblastoma cells [298,299]. TRIM32 is frequently upregulated in breast cancer, skin cancer, head and neck cancer and non-small lung cancer [117,267,268,269]. Also, TRIM59 is highly upregulated in breast, colorectal, gastric and pancreatic cancer and in several breast cancer and colorectal cancer cell lines, and this upregulation is correlated with lymph node metastasis in breast cancer and with poor prognosis for patients [78,270,271,272]. Mechanisms by which TRIM59 contributes to carcinogenesis include upregulation of glycolysis by the PI3K/AKT/mTOR pathway, suppression of the degradation of PDCD10 (programmed cell death protein 10) and downregulation of p53 [78,271,273].

The involvement of UBE3A (also known as E6AP) in viral oncogenesis was first established in HPV-associated cervical cancer [274]. Subsequent researches showed that UBE3A also plays a role in multiple cancers, including Burkitt’s lymphoma, prostate cancer, non-small cell lung cancer and breast cancer [122,275,276,277,278]. The molecular function of UBE3A in cancers depends, however, on specific tumor types. In a subset of prostate cancers, UBE3A mediates degradation of the tumor suppressor proteins PML and p27^Kip1^ and thus acts as an oncogene while in non-small cell lung cancer, UBE3A acts as a tumor suppressor [300,301]. In approximately 20% of non-small cell lung cancer UBE3A levels are decreased, resulting in lower levels of the cyclin-dependent kinase (CDK) inhibitor p16^INK4A^ and a reduction in overall survival [277]. In breast cancer, UBE3A appears to play conflicting roles and promotes and suppresses cancer progression by acting as a transcriptional co-activator for the estrogen receptor alpha, the progesterone receptor and the androgen receptor, and by ubiquitinating the estrogen receptor and androgen receptor and promoting their degradation [302].

UBE4B overexpression is often associated with amplification of its gene in human brain tumors, resulting most likely in inactivation of p53 in brain tumors [132]. UBE4B was also found upregulated in human primary hepatocellular carcinoma tissues and this amplified expression is correlated with poor outcome [279]. Silencing UBE4B by siRNA was associated with downregulation of Bcl-2 and upregulation of p53, Bax and cleaved-Caspase3 and resulted in inhibition of proliferation, colony formation, migration and invasion of hepatocellular carcinoma cells [279].

Like TOPORS, CHIP suppresses malignances. Accordingly, CHIP expression was found downregulated in esophageal squamous cell carcinoma, colorectal carcinoma, gastric cancer and breast cancer [280,281,282,303,304,305,306]. Enforced expression of CHIP reduced apoptosis and invasion, consequences that might arise from increased expression of the anti-apoptotic proteins Bcl-2 and Bcl-xL and a decrease in the expression of RelA (nuclear factor NF-kappa-B p65) and IKKβ (inhibitor of nuclear factor kappa-B kinase subunit beta), in addition to the regulation of p53 [127,280]. CHIP overexpression in gastric and colon cancer cells furthermore impeded the formation of anchorage independent colonies in soft agar, suppressed the growth of xenografts in nude mice and inhibited endothelial cell growth and angiogenesis [281,305]. In prostate cells, CHIP ubiquitinates the androgen receptor, a major driver of prostate cancer, and targets it for proteasomal degradation, indicating that CHIP might also be involved in prostate cancer [307]. CHIP is downregulated particularly in late stages of colorectal cancer when the CHIP promoter is hypermethylated. Decreased expression of CHIP in tumors correlates with an unfavorable prognosis and a shorter overall survival of cancer patients [282,305].

ARF-BP1 was found upregulated in breast cancer, and in about 40% of patients with a subset of liposarcoma that showed MDM2 overexpression [283]. For other E3s like TRIM39, MKRN1/2 Synoviolin or CARPs, less is published about their expression in human tumors or their prognostic value.

Thus, a large number of E3s that target p53 are deregulated in human cancers. However, as each of these ligases has several targets, and several of these targets may act as oncogenes or tumor suppressor proteins, it is difficult to judge how much of the tumor-promoting activity of the ligase is attributed to p53. Nevertheless, as p53 is such an important tumor suppressor protein, it is most likely that inactivation of p53 is a major driving force for the deregulation of these E3s in cancer.

## 9. Targeting E3s for Tumor Therapy

Given the fact that all the above described E3s affect p53’s transcriptional activity, it is a logic strategy to target these E3s for promoting tumor regression. An ideal anti-cancer target should meet five criteria: (i) it should play an essential role in carcinogenesis, and/or be required for the maintenance of a cancer cell phenotype, and/or render cancer cells resistant to apoptosis; (ii) it is activated or overexpressed in cancer cells and its activation or overexpression is associated with poor prognosis; (iii) its inhibition induces growth suppression and/or apoptosis in cancer cells; (iv) it can be easily screened for small molecular inhibitors by high-throughput methods; (v), it is either not expressed or only at a very low level in normal cells and its inhibition has minimal effects on normal cell growth and function [308]. All these criteria are provided by E3s that inactivate p53.

**Figure 8:**
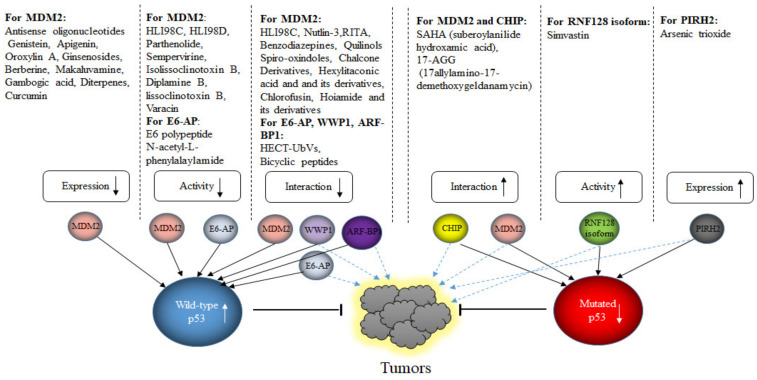
Strategies for E3s-dependent cancer therapy. Several strategies are used for targeting E3s. When tumor cells express wild-type p53, then p53 activity shall be enhanced. This can be achieved by reducing expression of the E3 (Strategy I), by reducing the ubiquitin ligase activity (Strategy II) or by preventing the interaction with p53 (Strategy III). For mutated p53, the most important strategy (Strategy I) is to release it from the interaction with the HSP90 complex, to make it accessible for E3s. A second strategy is to increase E3 activity and a third strategy to increase E3 abundance.

MDM2 is not only the most important E3 for p53, it also appears to be an ideal drug target (Figure 8). It is overexpressed in numerous cancer types and highly associated with poor prognosis, although the harm of its overexpression is not entirely uncontroversial [229,234]. First attempts to inactivate MDM2 were performed by knockdown experiments with MDM2 antisense-oligonucleotides which caused induction of p53 and p21 and inhibited proliferation of GEO human colon-cancer cells. Successive xenograft experiments showed anti-tumor activity of the MDM2 antisense oligonucleotides that was potentiated when they were combined with conventional cytotoxic drugs, such as cisplatin and topotecan [309], but also small molecules that inhibit MDM2 ubiquitin ligase activity were developed. HLI98C and HLI98D inhibited MDM2 ubiquitin ligase activity in in vitro experiments and caused p53-dependent apoptosis in cancer cells at micromolar concentrations [310]. Substantial attempts were made to inhibit the Mdm2/p53 interaction, particularly as the N-terminus of MDM2 forms a pocket into which the N-terminus of p53 inserts, providing an almost ideal structure for the development of small molecules that sit in the MDM2- pocket and displace p53 [16]. Nutlin-3, Benzodiazepines, Spiro-oxindoles and Quinolinols were found to displace p53 from the interaction with MDM2 and to increase p53 levels and activity in tumor cells [311,312,313,314]. Other compounds like Reactivation of p53 and Induction of Tumor cell Apoptosis (RITA) inhibit the interaction of p53 and MDM2 by binding to p53 [315].

More recently, a body of natural products, ranging from flavonoids, steroids and sesquiterpenes to alkaloids, have been explored as MDM2 inhibitors and shown to exhibited anti-tumor effects [316]. Parthenolide, sempervirine, isolissoclinotoxin B, diplamine B, lissoclinotoxin B, varacin are examples of inhibiters of MDM2 activity, while genistein, apigenin, oroxylin A, Ginsenosides, berberine, makaluvamine, gambogic acid, diterpenes and curcumin act by down-regulating MDM2 levels [317,318,319,320,321,322,323,324,325,326]. Several natural products such as chalcone derivatives, hexylitaconic acid and its derivatives, hoiamide and its derivatives and chlorofusin show anti-cancer activity by inhibiting the MDM2/p53 interaction [327,328,329,330].

Also, for HECT-domain E3s, targeting approaches have been developed (Figure 8). HECT-type E3s possess an N-terminal lobe for E2-binding and a C-terminal lobe with the active-site cysteine in their HECT domain [331]. In order to alter the activity of HECT-domain E3s, ubiquitin variants were designed that bind to the active-site cysteine or to the E2 binding site with greater affinity than wild-type ubiquitin, resulting in competitive inhibition of ubiquitination and/or suppression of ligase activity [332]. Bicyclic peptides have also been shown to inhibit the activity of HECT-type E3s including ARF-BP1 and WWP [333].

E3s can also be targeted to promote degradation of mutated p53 (Figure 8). Arsenic trioxide, for instance, induces expression of PIRH2 and thus degradation of mutated p53, and Simvastatin, a mevalonate inhibitor, promotes degradation of mutated p53 by promoting degradation of the ubiquitin ligase activity of RNF128 isoform 1 [216,217]. However, as mutated p53 is trapped in higher order complexes and thus largely protected from the access by E3s, therapeutic approaches focus more on possibilities to liberate mutated p53 from the higher order complexes to allow its degradation. One example for this is SAHA (suberoylanilide hydroxamic acid), an FDA (Food and Drug Administration)-approved histone deacetylase inhibitor. SAHA destabilizes the interaction of mutated p53 with HDAC6 and HSP90, which then allows MDM2 and CHIP to ubiquitinate the mutated p53 and target it for degradation [334]. Similarly, 17-AGG (17-allylamino-17-demethoxygeldanamycin), a HSP90 inhibitor liberates mutated p53 from the higher order complex with HDAC6 and HSP90 and allows MDM2- or CHIP-mediated degradation [334].

## 10. Discussion

The p53 tumor suppressor protein is a target of numerous E3s. The majority of them decorate p53 with ubiquitin and target it for proteasomal degradation while others modify p53 with SUMO or NEDD8. However, p53 does not seem to be a target for modification with ATG8, ATG12, URM1, UFM1, FAT10 or ISG15.

The large number of different E3s that promote p53 degradation raises the question why such a redundancy in E3s is necessary for controlling p53. One reason is certainly that not all E3s are expressed at the same time and in the same cell. MDM2, for example, is not expressed in liver, muscle and thymus on day 12.5 of embryonic development and on day 14.5 of mouse development not in colon epithelium, intestine, liver, muscle and salivary gland [335]. Thus, other E3s need to control p53 in these tissues during this time window. Other E3s appear to have very specific tasks during development or in certain tissues. TRIM71 is highly expressed during the period of neural tube closure and its loss results in aberrant p53 activation and aberrant neural tube development [81]. ARF-BP1 expression peaks at five to seven weeks of gestation and its expression is inversely correlated with that of p53 [336]. Other E3s are especially active in neurons, heart or muscle. UBE4B, for example is particularly important for preventing p53-mediated neuronal death and FBXO42, although detected in most human tissues, shows the highest expression in heart and skeletal muscle, suggesting that it has a prime function in these tissues [136,337]. Some E3s are correlated with certain settings, like CHIP with replicative senescence or synoviolin with the ER [66,67,224]. Several E3s are required to target p53 for degradation during the DNA damage response. After DNA damage, p53 and several E3s including MDM2 and PIRH2 are post-translationally modified [35,36,38,338]. These modifications allow the release of p53 from the interaction with these E3s and the stabilization of p53. However, after the repair of the lesion, p53 levels need to go back to normal. Therefore, other E3s that are not hindered in their interaction with p53 by post-translational modifications are required. E3s like CARPs also bind to post-translationally modified p53 [117]. Several of these E3s are induced after DNA damage and some are even p53 target genes [65,106]. The high level of these E3s after DNA damage help to get p53 levels back to normal once the lesions are repaired. Finally, it is possible that back-up systems have been installed to prevent inappropriate cell killing when one of the usual E3s drops out.

Of peculiarity are also the bi- and multifunctional E3s, that switch from a ligase for one ubiquitin-like molecule to an E3 for another one. Exchange of the F-Box protein FBXO42 of the SCF complex with the F-Box protein FBXO11 turns the SCF complex from a ubiquitin ligase for p53 to one that decorates p53 with NEDD8 [136,168]. While for this shift in activity an exchange of an integral part of the multi-subunit E3 is required, there are cases where one E3 can decorate p53 with two (TOPORS) or even three (MDM2) different ubiquitin-like proteins. For a few of them, it is already reported that post-translational modifications of the ligase can regulate the preference for one or another E2 thus shifting the ligase from an E3 for ubiquitin to one for SUMO [67,174]. It will be interesting to see whether more E3s can mate with several E2s, under which conditions this plays a role and how this is regulated at the molecular level.

While so many different E3s have been found for full-length p53, not many E3s have been reported for the degradation of p53 isoforms. One of the reasons may be that the isoforms are not known as long as the full-length protein, but more likely is, that the analysis of the isoforms is much more challenging and we are only at the beginning of having the right tools for the analysis of the p53 variants. The situation is further complicated as not only p53 comes in several isoforms but also several E3s express variants that occasionally also regulate the activity of the full-length E3 in addition to p53 [98,102,105].

Since p53 is an important tumor suppressor protein, it is less surprising that several of the E3 ligases that control p53 activity are also up- or downregulated during tumorigenesis and thus lead to a functional knock-out of p53 which may intermittently be the ultimate trigger for tumorigenesis. E3s are also possible drug targets. However, further research is needed to find windows that allow the control of the activity of these E3 in tumors without affecting adjacent healthy tissue.

## 11. Conclusions

p53 is a protein that obviously requires tight control. Therefore, a multitude of different E3s have been put in place to keep p53 in check e.g., when its activity is not wanted and would result in uncontrolled proliferation arrest and cell death. The large variety of E3s ensures that p53 is properly controlled in every cell, organ and conditions. The implementation of feed-back loops ensures a stable balance between the levels of p53 and the E3s. If this balance is lost, e.g., by uncontrolled under- or overexpression of the E3s, malformations can arise and diseases can develop. E3s are also attractive drug targets, however, windows may need to be defined to ensure the protection of healthy tissue.

## Figures and Tables

**Table 1:** E3s that target p53 for proteasomal degradation.

E3	Alias	Type	References
MDM2	Hdm2	RING	[55,56,57,58,59]
MKRN1	RNF61	RING	[60]
MKRN2	RNF62	RING	[61]
COP1	RNF200	RING	[62]
PIRH2	ZN363	RING	[63,64,65]
Synoviolin	HRD1	RING	[66,67]
TOPORS	P53BP3	RING	[68,69,70]
RNF1	RING1	RING	[71]
RNF2	RING1B	RING	[72,73]
RNF128	Grail	RING	[74]
TRIM24	TIF1A	RING	[75]
TRIM32	HT2A	RING	[38,76]
TRIM39	RNF23	RING	[77]
TRIM59	RNF104	RING	[78]
TRIM 69	RNF36	RING	[79,80]
TRIM71	Lin41	RING	[81]
CARP1/2	RNF34/RNF189	RING	[82]
UBE3A	E6-AP	HECT	[83,84,85,86]
ARF-BP1	HUWE1	HECT	[87,88]
CHIP	UBOX1	U-box	[89,90,91]
UBE4B	UBOX3	U-box	[92,93]

**Table 2:** E3s that mediate p53 ubiquitination for other purposes than targeting the tumor suppressor protein for degradation.

E3	Alias	Type	Impact on p53			References
			Nuclear export	Transcriptional activity	Protein stabilization	
MDM2	Hdm2	RING	√	√		[138,139]
MSL2	RNF184	RING	√			[140]
WWP1	AIP5	HECT	√	√	√	[141,142]
CUL7	KIAA0076	-		√		[143,144]
E4F1	E4F	RING		√		[145]

**Table 3:** E3s that mediate p53 sumoylation and/or neddylation.

E3s	Type	Sumoylation	Neddylation		Impact on p53		References
				Protein stabilization	Subcellular Localization	Transcriptional activity	
MDM2	RING	√	√	√		√	[157,158,163],
TOPORS	RING	√		√		√	[164]
PIAS-4	RING	√			√	√	[165,166,167]
FBXO11	F-Box		√		√	√	[168]

**Table 4:** E3s with altered expression in cancer.

E3	Modulation in Cancers	References
Mdm2	breast cancer, prostate cancer, colorectal adenocarcinoma, esophageal squamous cell carcinoma, gastric cancer, non-small cell lung cancer	[228,229,230,231,232,233]
COP1	breast adenocarcinomas, ovarian adenocarcinomas, pancreatic cancer, hepatocellular carcinoma, gastric cancer	[235,236,237]
PIRH2	hepatocellular carcinoma, head and neck cancer, prostate cancer, lung cancer, prostate cancer, ovarian cancer, breast cancer	[241,242,243,244]
RNF1	non-small cell lung cancer, hepatocellular cancer, prostate cancer	[245,246]
RNF2	colon cancer, gastric cancer B-cell lymphoma, Burkitt’s lymphoma, Hodgkin’s lymphomas, Melanoma	[247,248]
TOPORS	colon adenocarcinoma, breast cancer,	[249]
TRIM24	non-small cell lung cancer, chronic myeloid leukemia, head and neck cancer squamous cell carcinoma, glioma, gastric cancer, bladder cancer, hepatocellular carcinoma	[250,251,252,253,254,255,256]
TRIM25	endometrial cancer, ovary cancer, prostate cancer, lung cancer, breast cancer	[257,258,259,260,261]
TRIM28	gastric cancer, ovarian cancer, glioma, hepatocellular carcinoma, breast cancer	[262,263,264,265,266]
TRIM32	breast cancer, skin cancer, head and neck cancer, non-small cell lung cancer	[267,268,269,270]
TRIM59	colorectal cancer, gastric cancer, pancreatic cancer	[78,270,271,272,273]
UBE3A	HPV-associated cervical cancer, Burkitt’s lymphoma, prostate cancer, non-small cell lung cancer, breast cancer	[121,274,275,276,277,278]
UBE4B	hepatocellular carcinoma, brain tumors	[132,279]
CHIP	breast cancer, gastric cancer, colorectal cancer, esophageal squamous cell carcinoma	[280,281,282]
ARF-BP1	breast cancer, liposarcoma	[283]
